# Hsa_circ_0005273 facilitates breast cancer tumorigenesis by regulating YAP1-hippo signaling pathway

**DOI:** 10.1186/s13046-021-01830-z

**Published:** 2021-01-12

**Authors:** Xuehui Wang, Changle Ji, Jiashu Hu, Xiaochong Deng, Wenfang Zheng, Yunhe Yu, Kaiyao Hua, Xiqian Zhou, Lin Fang

**Affiliations:** 1 Department of Thyroid and Breast Surgery, Shanghai Tenth People’s Hospital, School of Medicine, Tongji University, Shanghai, 200072 China; 2 Nanjing Medical University, Nanjing, 211166 China

**Keywords:** hsa_circ_0005273, miR-200a-3p, YAP1, Hippo signaling pathway, Breast cancer

## Abstract

**Background:**

Circular RNAs (circRNAs), a novel class of endogenous RNAs, have shown to participate in the development of breast cancer (BC). Hsa_circ_0005273 is a circRNA generated from several exons of PTK2. However, the potential functional role of hsa_circ_0005273 in BC remains largely unknown. Here we aim to evaluate the role of hsa_circ_0005273 in BC.

**Methods:**

The expression level of hsa_circ_0005273 and miR-200a-3p were examined by RT-qPCR in BC tissues and cell lines. The effect of knocking down hsa_circ_0005273 in BC cell lines were evaluated by examinations of cell proliferation, migration and cell cycle. In addition, xenografts experiment in nude mice were performed to evaluate the effect of hsa_circ_0005273 in BC. RNA immunoprecipitation assay, RNA probe pull-down assay, luciferase reporter assay and fluorescence in situ hybridization were conducted to confirm the relationship between hsa_circ_0005273, miR-200a-3p and YAP1.

**Results:**

Hsa_circ_0005273 is over-expressed in BC tissues and cell lines, whereas miR-200a-3p expression is repressed. Depletion of hsa_circ_0005273 inhibited the progression of BC cells in vitro and in vivo, while overexpression of hsa_circ_0005273 exhibited the opposite effect. Importantly, hsa_circ_0005273 upregulated YAP1 expression and inactivated Hippo pathway via sponging miR-200a-3p to promote BC progression.

**Conclusions:**

Hsa_circ_0005273 regulates the miR-200a-3p/YAP1 axis and inactivates Hippo signaling pathway to promote BC progression, which may become a potential biomarker and therapeutic target.

**Supplementary Information:**

The online version contains supplementary material available at 10.1186/s13046-021-01830-z.

## Background

Breast cancer (BC) is the most commonly diagnosed malignancy and the leading cause of cancer-related deaths in women [1]. Despite robust advances in surgical and medical management in BC, the morbidity and mortality are not decreasing [2]. Higher rates of metastasis, recurrence and drug resistance are the significant reasons of cancer-associated deaths among breast cancer patients. Therefore, further investigating the molecular mechanism of BC remains an urgent and difficult task.

Circular RNAs (circRNAs), a novel class of endogenous non-coding RNAs (ncRNAs), are formed from exons or introns through special selective shearing [3]. Unlike linear RNAs that are terminated with 5’caps and 3’tails, circRNAs are single-stranded covalently closed circular transcripts [4, 5], providing them higher ability to resist to environmental degradation [6]. Recent years, circRNAs have been proved as diagnostic biomarkers of hepatoblastoma [7] and bladder cancer [8] in blood or plasma according to their stability and tissue specificity [9, 10]. Aberrant expression of circRNAs are correlated with the progression and prognosis of various cancers [11]. Emerging evidence illustrated that circRNAs play key oncogenic or anti-cancer roles in multiple cancers, including BC. For example, circRNA_0025202 regulates tamoxifen sensitivity and tumor progression via regulating FOXO3a [12]. CircFBXW7 inhibits the progression of TNBC through encoding a 185-aa Protein [13]. Therefore, the potential function and molecular mechanisms of circRNAs in BC need further investigation.

Hippo signaling pathway, a highly conserved pathway, performs an important role in governing cell proliferation and apoptosis of various cancer types, especially in BC. The dysregulation and inactivation of Hippo pathway always result in cancer initiation and progression [14]. YAP1 (yes-associated protein), a downstream gene of Hippo signaling pathway, is a significant oncogene in BC [15, 16]. Inhibiting the activity of YAP1 could restrain vascular invasiveness of BC cells [17]. Since the mutation, activation and overexpression of YAP1 are related to the occurrence and progression of BC, finding a new molecule that could suppress YAP1 is imperative.

In this research, we identified hsa_circ_0005273, a circRNA of Protein Tyrosine Kinase 2 (PTK2), was significantly high-expressed in BC tissues and cell lines. Importantly, hsa_circ_0005273 exerted its oncogenic role in BC via acting as a sponge of tumor suppressor miR-200a-3p to inactivate YAP1-Hippo signaling pathway in vivo and in vitro. Our findings provided new insights into the diagnosis and treatment of BC.

## Materials and methods

### CircRNA expression profiling analysis

CircRNAs expression dataset of BC were downloaded from NCBI GEO database (https://www.ncbi.nlm.nih.gov/geo/) and analyzed using the GEO2R (http://www.ncbi.nlm.nih.gov/geo/geo2r/) to show different circRNAs expression.

### Tissue samples

Tumor tissues and their adjacent normal tissues of 120 BC patients were collected from the Department of Breast and Thyroid Surgery of Shanghai Tenth People’s Hospital of Tongji University (Shanghai, China). None of the patients received any local or systemic treatment before surgery and all tissue specimens were immediately snap-frozen in liquid nitrogen until further use. Our study protocols were approved by Institutional Ethics Committees of Shanghai Tenth People’s Hospital and informed consent was obtained from all patients or their relatives. The methodology of this study adhered to the standards outlined in the Declaration of Helsinki.

### Cell culture and transfection

The human BC cell lines MDA-MB-231, MCF-7, HCC-1937, SKBR3 and normal breast epithelial cell line MCF-10A were purchased from Chinese Academy of Sciences (Shanghai, China). MDA-MB-231, MCF-7, HCC-1937 and SKBR3 cells were cultured in Dulbecco’s Modified Eagle’s Medium (DMEM) (Gibco, USA) with 10% Fetal Bovine Serum (FBS) (Gibco, USA), penicillin (100 units/ml) and streptomycin (100 μg/ml) (Enpromise, China). MCF-10A cells were cultured in Mammary Epithelial Basal Medium (MEBM) (Cambrex, USA). All these cells were cultured at 37 °C with 5% CO2. Small, interfering, specifically targeting human hsa_circ_0005273 (si-circ_0005273), non-specific negative control oligos (si-NC) and hsa_circ_0005273 lentiviral plasmid (lv-circ_0005273) were purchased from IBSBio (Shanghai, China). Human miR-200a-3p-mimics, non-specific negative control (miR-200a-3p-NC) and miR-200a-3p inhibitor were purchased from RiboBio (Guangzhou, China). MDA-MB-231, MCF-7 and SKBR3 cells were cultured and transfected with reagents above using Lipofectamine® 2000 (Invitrogen; Thermo Fisher Scientific, USA) according to the manufacturer’s instructions. We used DNA Midiprep Kits (Qiagen, Hilden, Germany) to prepare plasmid vectors. A lentivirus carrying si-circ_0005273 was constructed by ZORIN (Shanghai, China) and transfection procedures were performed according to the manufacturer’s instructions.

### RNA extraction and RT-qPCR

Total RNA was extracted from frozen tissues and cultured cells by Trizol reagent (Invitrogen, Carlsbad, CA, USA) and the concentration and purity of RNA samples was assessed with a Nanodrop 2000 spectrophotometer (Thermo Fisher Scientific, USA). cDNA was synthesized by a commercial cDNA synthesis kit (Takara Biotechnology, Dalian, China). We conducted quantitative real-time polymerase chain reaction (RT-qPCR) by using the SYBR Green PCR Kit (Takara Biotechnology, Dalian, China) and primer sequences were designed and synthesized by RiboBio (Guangzhou, China). Expression of circRNA, miRNA and mRNA were assessed by threshold cycle (CT) values and analyzed using the 2^-ΔΔCt^ method. Primers and siRNAs designed in this study are shown in supplemental Table S1.

### Confirming specificity for hsa_circ_0005273

PCR products amplified by primers were separated on 1% agarose gel to verify the specificity of the hsa_circ_0005273 PCR products. Sanger sequencing was performed to validate the sequence of hsa_circ_0005273.

### RNase R resistance analysis of circRNAs

MDA-MB-231, MCF-7 and SKBR3 cell lines were treated with RNase R (4 U/mg, Epicenter) and incubated for 30 min at 37 °C. Then, the treated RNAs were reverse transcribed with specific primers and detected by RT-qPCR assay.

### MTT assay

A density of 2000 cells per well were placed into 96-well plates. The cells were detected in accordance with the manufacturer’s instructions using MTT assay kit (Sigma, Santa Clara, CA, USA). The 490 nm optical density was detected by a microplate reader respectively at 24, 48, 72 and 96 h.

### Colony formation assay

A density of 1000 cells per well were transferred into six-well plates. Cell colonies were washed twice by using cold phosphate buffered saline (PBS), fixed with 75% ethanol and stained with 0.1% crystalline purple until the colonies were visible. Then colonies were counted and photographed.

### Wound healing assay

MDA-MB-231, MCF-7 and SKBR3 cells were transfected with a range of constructs as indicated in 6-well plates. When the treated cells reached about 80% confluent, a scratch was produced in the cell monolayer by drawing a 200-μl-pipette tip over the surface of each well, holding the tip perpendicular to the plate. The monolayers were washed twice with 1x PBS and cultured with DMEM medium with 2%FBS. Wound healing was observed under a light microscope and pictures were taken at 0 h, 24 h and 48 h at the same position to observe cell movement.

### Migration assays

Transwell chambers (Corning, Inc., Lowell, MA, USA) were used to measure the migration ability of the cells in 24-well plates. Cells were transferred into the upper chamber with 200 μl serum-free medium and medium with 10% FBS was added to the lower chamber. 24 h later, cells in the upper chamber were carefully removed by a cotton swab. Then, the cells on the opposite side of the filter were fixed with 70% ethanol for 30 min and stained with 0.1% crystal violet for 10 min. Representative pictures were taken with a microscope (Leica Microsystems, Mannheim, Germany) and migrated cells were counted in five random fields.

### Cell cycle assay

MDA-MB-231, MCF-7 and SKBR3 cells were transfected with a range of constructs in 12-well plates. Cells were collected and fixed in ice-cold ethanol for more than 4 h. Then each sample was added with 0.5 ml 0.05 mg/ml propidium iodide (PI) staining solution and incubated for 30 min at 37 °C. Flow cytometer (FACSCantoTM II, BD Biosciences) was used to analyze the cell cycle .

### Dual-luciferase reporter assay

To confirm that miR-200a-3p directly targets hsa_circ_0005273 and YAP1 3′-UTR, wild and mutant reporter plasmids of hsa_circ_0005273 and YAP1 were individually designed and synthesized by IBSBio (Shanghai, China). 293 T cells were co-transected with the constructed reporter plasmids, together with miR-200a-3p mimics or miR-200a-3p-NC using Lipofectamine® 2000 (Invitrogen; Thermo Fisher Scientific, USA). 24 h later, luciferase activities were measured by the Dual-Luciferase® Reporter Assay kit (Promega, Madison, USA). Then firefly to Renilla luciferase ratios were calculated.

### RNA antisense purification (RAP)

Approximately 6 × 10^7^ MDA-MB-231 cells were washed with ice-cold 1 × PBS, lysed in lysis buffer and incubated for 10 min at 37 °C with DNase salt stock. 100 ul high-affinity biotin RNA probe was added into RAP samples, hybridized at 37 °C for 30 min, incubated at 50 °C for 5 min, then hybridized at 37 °C for 2 h. Streptavidin beads and RAP samples were incubated for 30 min at room temperature and washed for 5 times with 500 μl washing buffer. After elution and purification, the RNA was restored by 30 μl RNase-free water analyzed by qRT-PCR. Hsa_circ_0005273 probe was designed by IBSBio (Shanghai, China) and RNA Antisense Purification (RAP) Kit was purchased from BersinBioTM (Guangzhou, China).

### RNA Immunoprecipitation assay (RIP)

RIP assay was conducted in MDA-MB-231 cells using the BersinBioTM RNA-Binding Protein Immunoprecipitation Kit (Guangzhou, China) according to manufacturer’s instructions. Anti-AGO2(Abclonal, China) and anti-IgG(Abclonal, China) were used. The extracted RNAs were analyzed by RT-qPCR.

### Western blotting analysis

Proteins were extracted by using RIPA lysis buffer (Beyotime, Jiangsu, China) and the concentrations were detected by using the protein assay kit (Beyotime, Jiangsu, China). Protein lysates were separated by 10% sodium dodecyl sulfate-polyacrylamide gels and then transferred to nitrocellulose membrane (Beyotime, Jiangsu, China), which was incubated 1 h with 5% non-fat milk and immunoblotted overnight at 4 °C with primary antibodies: anti-PCNA(Proteintech, USA), anti-YAP1(Abclonal, China), anti-p-YAP1(Abclonal, China), anti-MST1(Abclonal, China), anti-p-MST1(Sigma, USA), anti-LATS1/2(Abclonal, China), anti-p-LATS1/2(Abclonal, China), anti-PTK2(Abclonal, China), anti-β-actin(Abclonal, China), and anti-LaminA(Proteintech, USA). The next day, the membranes were incubated in secondary antibodies for 1 h at room temperature. Dilutions of all antibodies used in this study were 1/1000. Signals of protein bands were scanned by Odyssey Infrared scanning system (Li-Cor, Lincoln, NE, USA).

### FISH assay

Ribo™ Fluorescent In Situ Hybridization Kit (Ribo, China) was used in FISH assay. Specific probes for the hsa_circ_0005273 were designed and synthesized by RiboBio (Guangzhou, China) and specific probes for the miR-200a-3p were designed and synthesized by IBSBio (Shanghai, China). 4′,6-Diamidino-2-Phenylindole (DAPI) was used to stain cell nuclei. Fluorescence microscope (Olympus BX53 Biological Microscope) was used to capture the images of cells.

### Xenograft tumor assay

Athymic nude mice (age, 4–6 weeks; weight, 18–22 g) were ordered from the laboratory animal center of Shanghai. Approximately 1 × 10^6^ MDA-MB-231 cells with stable expression of si-circ_0005273 or si-NC were injected into the second mammary fat of the mice (*n* = 4, each group). Then, tumor size was measured and calculated every week using the following formula: Volume (mm^3^) = 0.5 * width^2^ * length. After 5 weeks, the mice were killed by cervical dislocation and the tumors were collected. The animal protocols complied with the rule of the ethics committee of Tongji University.

### Immunohistochemistry (IHC)

Fresh tumor tissue samples from the nude mice were fixed in 4% paraformaldehyde, hydrated through ethanol solution and embedded in paraffin. The paraffin-embedded tissue was sectioned into 4 μm slides, then the sections were incubated with anti-YAP1, anti-MST1 and anti-LATS1/2 antibodies to measure YAP1, MST1 and LATS1/2 expression. Images were captured under a microscope (Leica Microsystems, Mannheim, Germany) at the appropriate magnification.

### Statistical analysis

The significance of differences between groups was assessed by GraphPad Prism V8.0 (GraphPad, CA, USA) and SPSS 20.0 (IBM, SPSS, IL, USA). Comparisons between groups were analyzed with the Student’s t test. Data were obtained from three independent experiments which are presented as the means ± standard deviation (SD) and a *P*-value < 0.05 was considered significant.

## Results

### The characteristic of hsa_circ_0005273 in BC cells

**Fig. 1:**
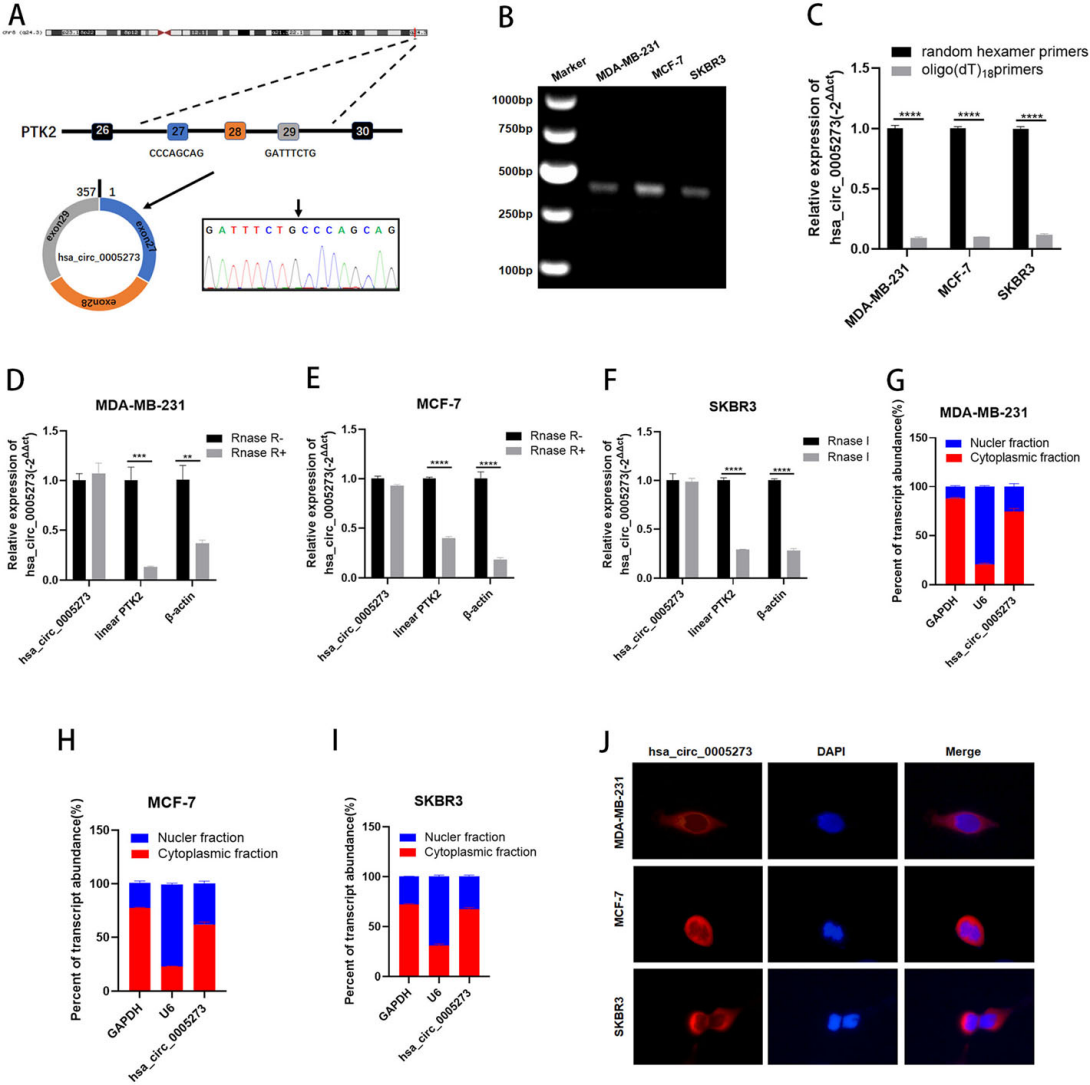
The characteristic of hsa_circ_0005273 in BC cells. **A** Hsa_circ_0005273 is formed by circularization of exon 27–29 of the gene PTK2, and the splicing junction was verified by Sanger sequencing. **B** Existence of hsa_circ_0005273 in BC cells was verified by agarose gel electrophoresis. **C** Expression of hsa_circ_0005273 in BC cells with random hexamer or oligo (dT)18 primers. **D-F** RT-qPCR analysis of hsa_circ_0005273, linear PTK2 and β-actin in BC cells treated with RNase R. **G-I** Expression levels of cytoplasmic control transcripts (GAPDH), the nuclear control transcript (U6), and hsa_circ_0005273 were determined by RT-qPCR in the cytoplasmic and nuclear fractions of BC cells. **J** RNA FISH for hsa_circ_0005273 and nuclei was stained with DAPI. Red, hsa_circ_0005273; Blue, DAPI. *p < 0.05, **p < 0.01,*** *p* < 0.001,**** *p* < 0.0001

GSE113230 was a dataset of non-coding RNA profiling by high throughput sequencing in triple negative BC (https://www.ncbi.nlm.nih.gov/geo/). We analyzed the dataset and found that the expression of a novel circRNA, termed hsa_circ_0005273, was higher in BC tissues than in adjacent normal tissues (Fig. S1A). In accordance with the UCSC Genome Browser Home (http://genome.ucsc.edu/), the 357-bp-long hsa_circ_0005273 is formed by circularization of exon 27–29 of the gene PTK2, which is located at chromosome 8:141710989–141,716,304. The hsa_circ_0005273 back splicing junction was verified by Sanger sequencing (Fig. 1a). To validate the existence of hsa_circ_0005273 in BC cells, we examined the PCR products amplified by primers on 1% agarose gel. Only a single band was observed in the gel and the PCR product was considered to be specific (Fig. 1b). Since circRNAs have no 3’poly-A tail due to the closed loop structures, we used both random hexamer and oligo (dT)18 primers in BC cells. The results showed that the relative expression of hsa_circ_0005273 was significantly downregulated when oligo (dT)18 primers were used compared with random hexamer primers, proving that hsa_circ_0005273 has no 3’ploy-A tail (Fig. 1c). Furthermore, RNase R exonuclease was used to further validate hsa_circ_0005273 in MDA-MB-231, MCF-7 and SKBR3 cell. Resistance to RNase R exonuclease confirmed that hsa_circ_0005273 was indeed circular (Fig. 1d-f). To explore the cellular distribution of hsa_circ_0005273, the expression levels of cytoplasmic control transcripts GAPDH, nuclear control transcript U6 and hsa_circ_0005273 were examined by RT-qPCR in the cytoplasmic and nuclear fractions of MDA-MB-231, MCF-7 and SKBR3 cell lines. Subcellular fractionation demonstrated quantitatively that hsa_circ_0005273 was enriched in the cytoplasm of three cell lines above (Fig. 1g-i). RNA fluorescence in situ hybridization (FISH) analysis also revealed that hsa_circ_0005273 was mostly stained in cytoplasm of BC cell lines (Fig. 1j).

### Hsa_circ_0005273 is highly expressed in BC and exerts oncogenic effects in BC cells

**Fig. 2:**
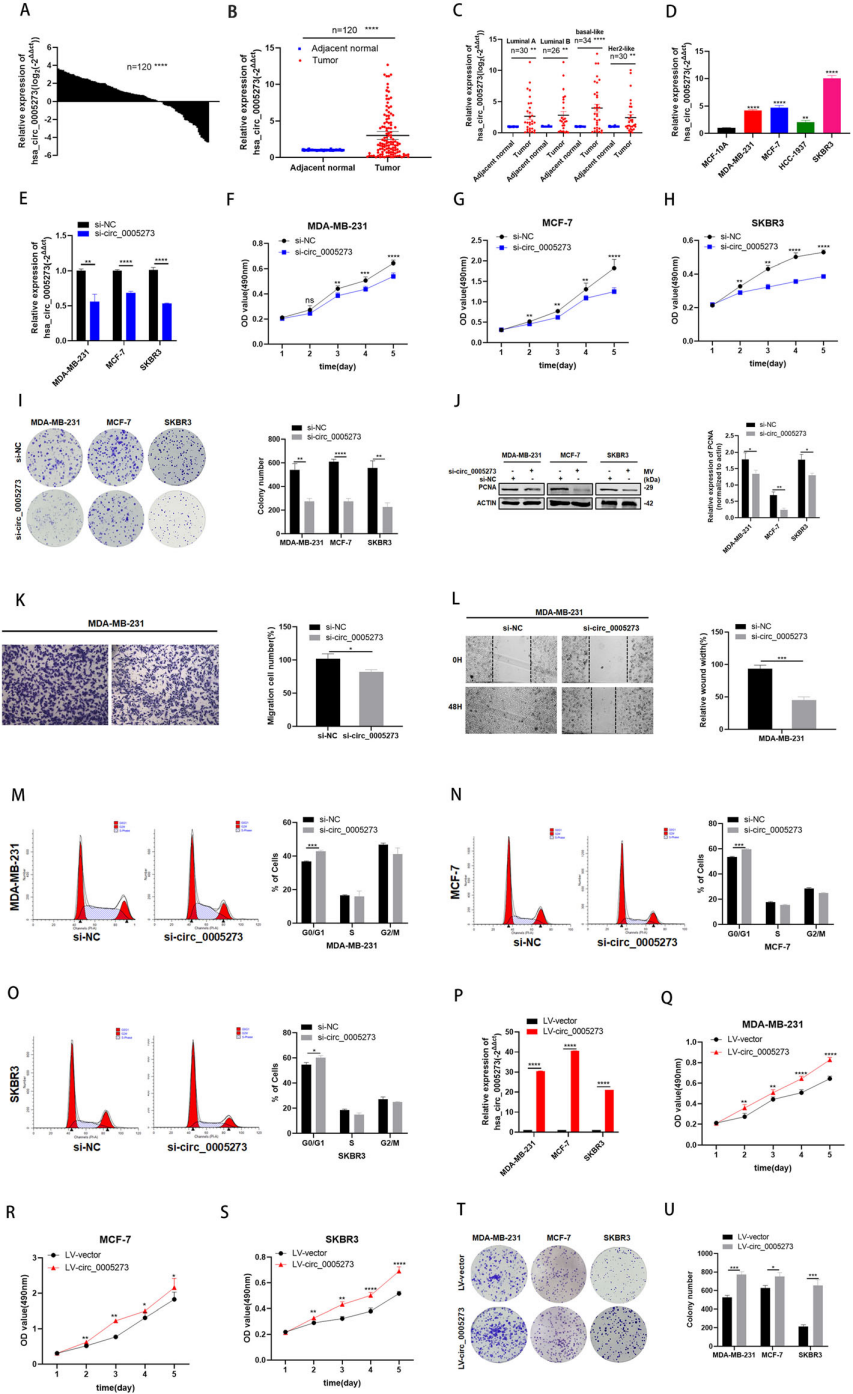
Hsa_circ_0005273 is highly expressed in BC and exerts oncogenic effects in BC cells. **A-B** Hsa_circ_0005273 was highly expressed in tumor tissues compared with adjacent normal tissues. **C** Expression of hsa_circ_0005273 in basal-like cohort, Her2-like cohort, luminal-A and luminal-B cohort, respectively. **D** Relative expression of hsa_circ_0005273 in BC cell lines. **E** Expression of hsa_circ_0005273 was confirmed by RT-qPCR in BC cells transfected with si-NC or si-circ_0005273. **F-H** Effect of si-circ_0005273 on proliferation in BC cells by MTT assay. **I** Effect of si-circ_0005273 on proliferation in BC cels by colony formation assay. **J** Effect of si-circ_0005273 on proliferation in BC cells by western blotting. **K** Cell migration assays were performed in MDA-MB-231 using transwell chambers. **L** Wound healing assays were performed in MDA-MB-231 treated with si-circ_0005273. **M-O** Cell cycle assays were performed in BC cells treated with si-circ_0005273. **P** Expression of hsa_circ_0005273 was confirmed by RT-qPCR in BC cells transfected with LV-vtctor or LV-circ_0005273. **Q-S** Effect of LV-circ_0005273 on proliferation in BC cell lines by MTT assay. **T** Effect of LV-circ_0005273 on proliferation in BC cell lines by colony formation assay. *p < 0.05, **p < 0.01,*** *p* < 0.001,**** *p* < 0.0001

The expression of hsa_circ_0005273 was assessed by RT-qPCR in 120 pairs of BC tissues and adjacent normal tissues, and our results showed that the expression of hsa_circ_0005273 was significantly elevated in BC tissues (83/120, 69.2%) compared with adjacent normal tissues (Fig. 2a and b). Based on American Joint Committee on Cancer eighth edition, 120 BC patients were divided into four cohorts, including basal-like, Her2-like and luminal-A and luminal-B. Hsa_circ_0005273 was significantly high expressed in basal-like cohort (24/34, 70.6%), Her2-like cohort (22/30, 73.3%), luminal-A cohort (19/30, 63.3%) and luminal-B cohort (18/26, 69.2%) (Fig. 2c). In addition, the expression of hsa_circ_0005273 was significantly increased in 4 BC cell lines (MDA-MB-231, MCF-7, HCC-1937 and SKBR3) compared with the normal breast epithelial cell line MCF-10A (Fig. 2d). Elevated expression of hsa_circ_0005273 in BC tissues reflected its tumor-promoting role in BC. To investigate the function of hsa_circ_0005273 in BC, the expression of hsa_circ_0005273 in the MDA-MB-231, MCF-7 and SKBR3 cell lines was depleted using a specific siRNA (si-circ_0005273) and si-NC was taken as a control. The transfection efficiency was verified by RT-qPCR (Fig. 2e). As expected, MTT assays and colony formation assays demonstrated that hsa_circ_0005273 depletion decreased cell proliferation in MDA-MB-231, MCF-7 and SKBR3 cells (Fig. 2f-i). Results from western blotting analysis demonstrated that expression of proliferation marker PCNA was inhibited by hsa_circ_0005273 siRNAs (Fig. 2j). Moreover, through transwell migration assays, hsa_circ_0005273 depletion decreased cell migration in MDA-MB-231(Fig. 2k). Consistently, limited migration was seen in the hsa_circ_0005273 low-expression group compared to the controls in wound healing after 48 h (Fig. 2l, S1B-D). Flow cytometry analysis showed that the percentage of G0/G1 phase cells increased in hsa_circ_0005273 depletion group (Fig. 2m-o). For further verification, we stably overexpressed more than 30-fold hsa_circ_0005273 in BC cells (Fig. 2p). Then, MTT assays and colony formation assays were performed to examine the effect of hsa_circ_0005273 overexpression. We found that upregulation of hsa_circ_0005273 significantly promoted BC cells proliferation (Fig. 2q-u). In addition, we analyzed the relationship between the expression of hsa_circ_0005273 and the clinical pathological variables in 120 BC patients. We found that high expression of hsa_circ_0005273 was positively associated with TNM stage, lymph node metastasis, tumor size and distant metastasis, but had no correlation with age (Table 1). Furthermore, relationship between the expression of hsa_circ_0005273 and the clinical pathological variables in three pathological groups have also been analyzed (Table S2, S3 and S4).

**Table 1:** The relationship between the expression of hsa_circ_0005273 and various clinicopathological variables in BC patients

Patients Characteristics	Total	hsa_circ_0005273 expression		
		High (*N = 83)*	Low (*N = 37)*	*P* value*
Age				0.7933
< 60	53	36	17	
≥ 60	67	47	20	
TNM stage				0.0030**
I and II	60	34	26	
III and IV	60	49	11	
Tumor size (cm)				0.0029**
≤ 2	70	41	29	
>2	50	42	8	
Lymph node metastasis				0.0151*
negative	82	51	31	
positive	38	32	6	
Distant metastasis				0.0057**
No	105	68	37	
Yes	15	15	0	

* p < 0.05, **p < 0.01

### Hsa_circ_0005273 serves as a sponge for miR-200a-3p

**Fig. 3:**
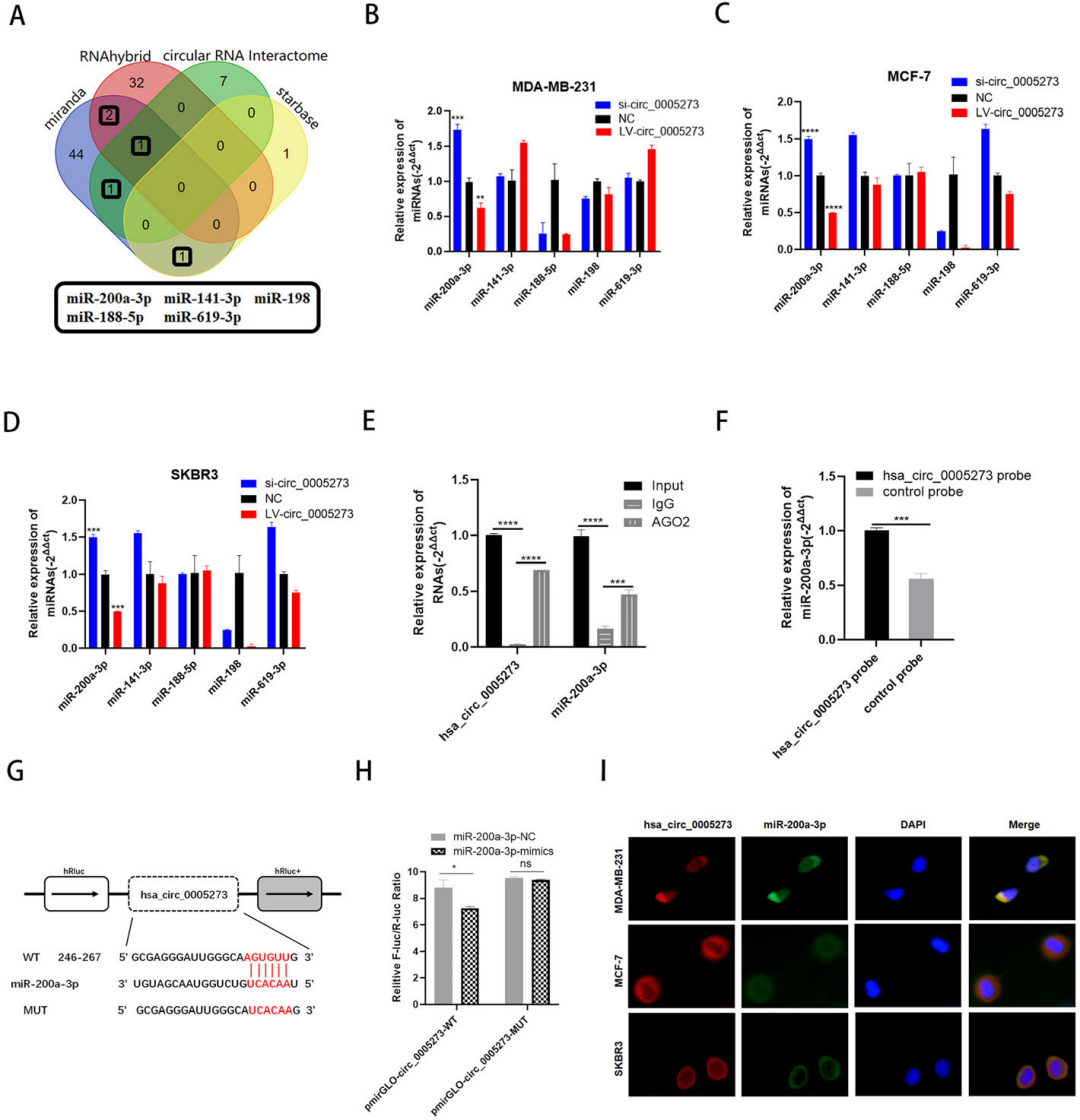
Hsa_circ_0005273 serves as a sponge for miR-200a-3p. **A** Venn diagram showing the potential target miRNAs of hsa_circ_0005273. **B-D** Expression of miRNAs in BC cells transfected with si-circ_0005273 or LV-circ_0005273. **E** RIP experiments were performed in HEK293T cells, and the co-precipitated RNA was subjected to RT-qPCR for hsa_circ_0005273 and miR-200a-3p. **F** MiR-200a-3p was pulled down and enriched with hsa_circ_0005273 specific probe and then detected by RT-qPCR. **G** Putative complementary sites within miR-200a-3p and hsa_circ_0005273 predicted by bioinformatics analysis (starbase). **H** Dual luciferase reporter assays demonstrated that miR-200a-3p is a direct target of hsa_circ_0005273. **I** Detection of colocalization of hsa_circ_0005273 and miR-200a-3p in cytoplasm by RNA FISH assay (magnification, × 400). Green, miR-200a-3p; Red, hsa_circ_0005273; Blue, DAPI. *p < 0.05, **p < 0.01,*** *p* < 0.001,**** *p* < 0.0001

To explore the function of hsa_circ_0005273, we first tested whether hsa_circ_0005273 could affect the expression of its parental gene PTK2. Results of RT-qPCR and western blotting showed that hsa_circ_0005273 had no influence on the transcription and translation of PTK2(Fig. S1E,F). Since the function of circRNA was related to the subcellular localization and hsa_circ_0005273 was mainly located in the cytoplasm of BC cells, we hypothesized that hsa_circ_0005273 exert oncogenic function through acting as a ceRNA in BC cells. Starbase (http://starbase.sysu.edu.cn/index.php), CircInteractome (https://circinteractome.nia.nih.gov/), miranda and RNAhybird were employed to predict potential miRNA binding partners of hsa_circ_0005273. With the help of Venn diagram, we chose 5 miRNAs(miR-200a-3p, miR-141-3p, miR-188-5p, miR-198, miR-619-3p) (Fig. 3a). The results of RT-qPCR showed that only the expression of miR-200a-3p performed negative correlation with hsa_circ_0005273 expression in MDA-MB-231, MCF-7 and SKBR3 cells, suggesting that hsa_circ_0005273 could bind to miR-200a-3p(Fig. 3b-d). RNA immunoprecipitation (RIP) assay was first performed to determine the association between hsa_circ_0005273 and miR-200a-3p. The results of RT-qPCR showed that the expression of both hsa_circ_0005273 and miR-200a-3p pulled down with anti-AGO2 was significantly higher compared to the anti-IgG, suggesting that hsa_circ_0005273 could directly target miR-200a-3p in an AGO2 manner (Fig. 3e). Then, compared with the control group, specific enrichment of miR-200a-3p was detected in the hsa_circ_0005273 pull-down pellet (Fig. 3f). Additionally, as predicted in starbase, there was a complementary base sequence between hsa_circ_0005273 and miR-200a-3p(Fig. 3g). To validate whether miR-200a-3p could directly target hsa_circ_0005273, we performed dual luciferase assays. Luciferase reporters indicated that miR-200a-3p significantly inhibited the luciferase reporter activity compared with the miRNA mimic negative control, suggesting that hsa_circ_0005273 could bind to miR-200a-3p(Fig. 3h). Additionally, FISH analysis results showed that hsa_circ_0005273 and miR-200a-3p were co-localized in the cytoplasm of BC cells (Fig. 3i).

### MiR-200a-3p is expressed at low levels and acts as a tumor suppressor in BC cells

**Fig. 4:**
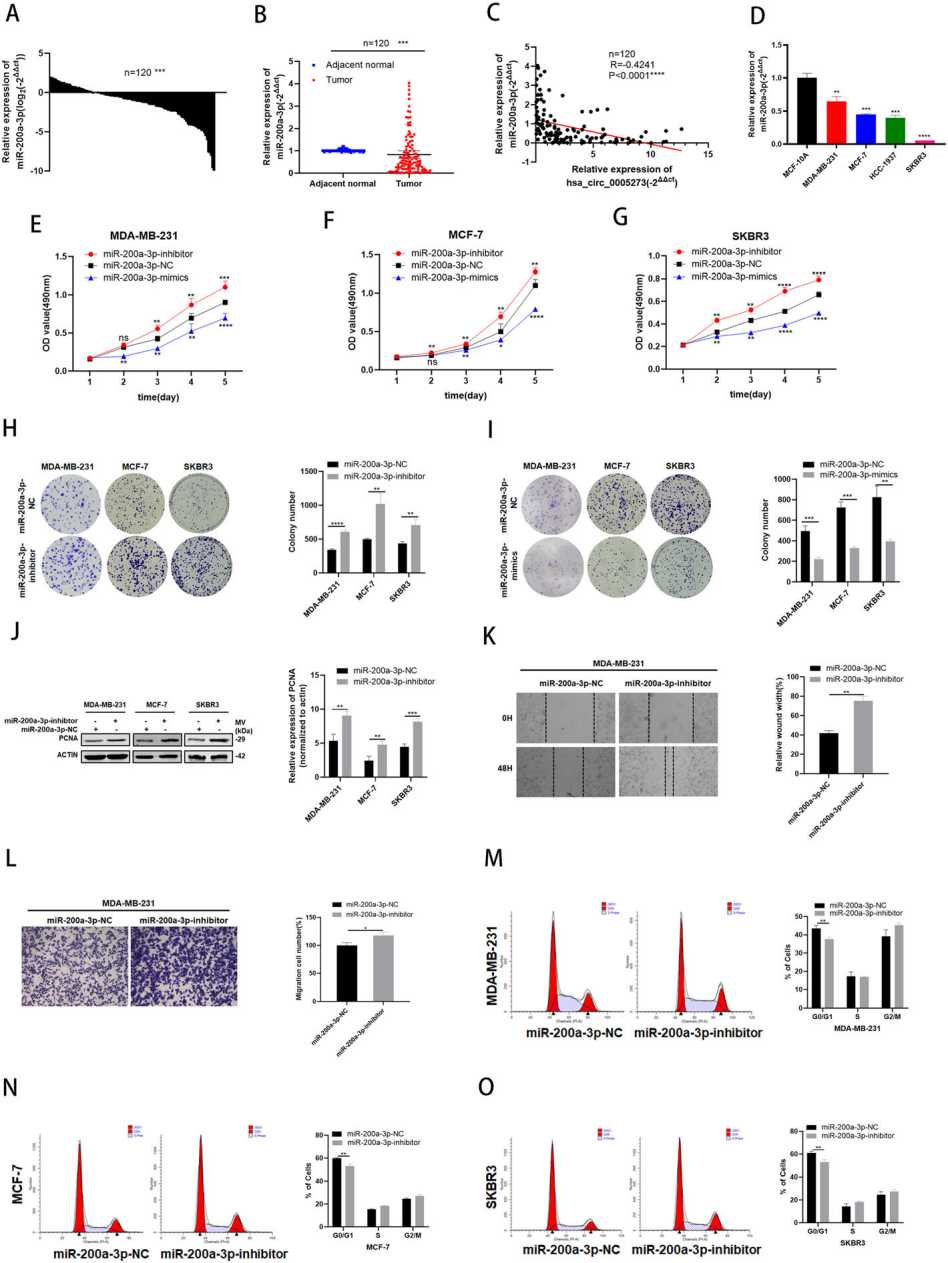
MiR-200a-3p is expressed at low levels and acts as a tumor suppressor in BC cells. **A-B** MiR-200a-3p had low expression in BC tissues compared with adjacent normal tissues. **C** Correlations between the expression of hsa_circ_0005273 and miR-200a-3p were found with Pearson’s correlation analysis in BC tissue samples (*n* = 120). **D** MiR-200a-3p had low expression in BC cell lines. **E-G** Effect of miR-200a-3p-mimics on proliferation in BC cells by MTT formation assay. **H-I** Effect of miR-200a-3p-inhibitor on proliferation in BC cells by colony formation assay. **J** Effect of miR-200a-3p-inhibitor on proliferation in BC cells by western blotting. **K** Wound healing assays were performed in MDA-MB-231 cell treated with miR-200a-3p-inhibitor. **L** Cell migration assays were performed in MDA-MB-231 cells using Transwell chambers. **M-O** Cell cycle assays were performed in BC cells treated with miR-200a-3p-inhibitor. *p < 0.05, **p < 0.01,*** *p* < 0.001,**** *p* < 0.0001

In order to explore the role of miR-200a-3p in BC, the expression level of miR-200a-3p was detected. The results of RT-qPCR demonstrated that miR-200a-3p presented with low expression in BC patient tissues and BC cell lines (Fig. 4a,b,d). Negative correlations between the expression of hsa_circ_0005273 and miR-200a-3p were found with Pearson’s correlation analysis in 120 BC tissue samples (Fig. 4c). The effects of miR-200a-3p on the proliferation ability of BC cells were tested in MTT assays, colony formation assays and western blotting (Fig. 4e-j). The effects of miR-200a-3p on the migration ability of BC cells were tested in Wound healing assays and transwell chambers (Fig. 4k,l). Moreover, results of cell cycle assays revealed that G0/G1 phase arrest can be initiated by miR-200a-3p(Fig. 4m-o). Results above suggested that miR-200a-3p could suppress the tumorigenesis of BC cells.

### MiR-200a-3p directly target YAP1

**Fig. 5:**
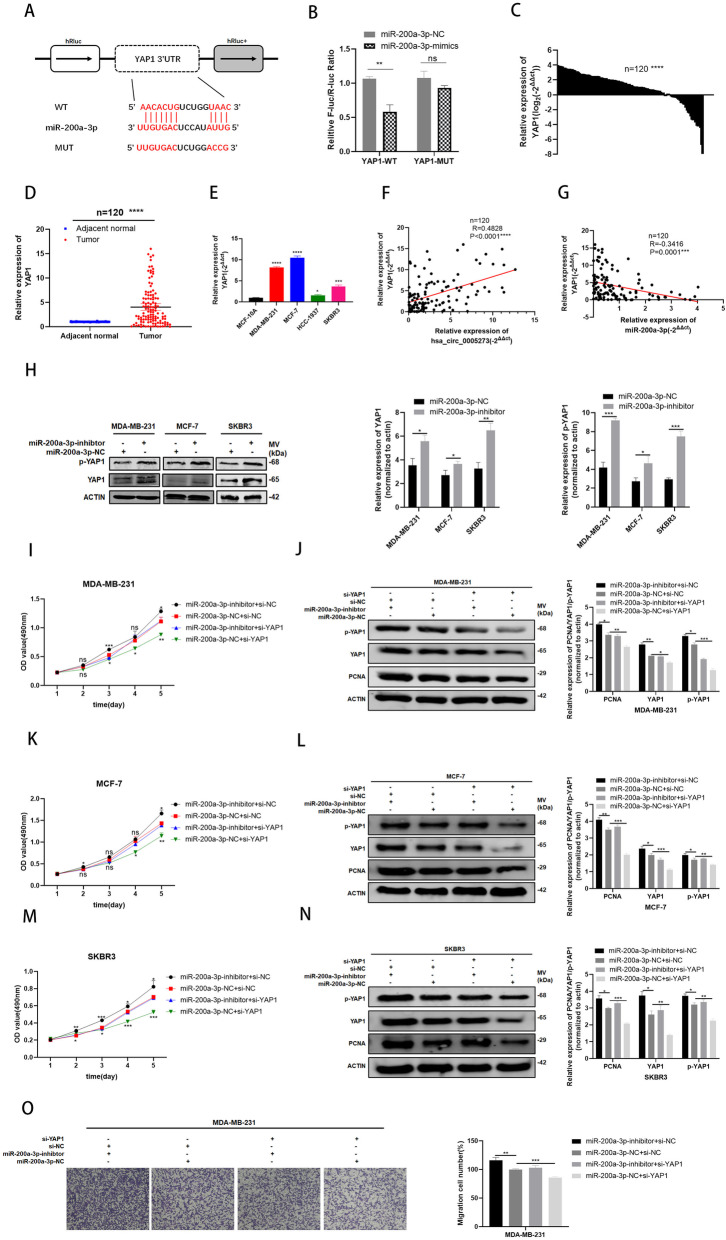
MiR-200a-3p directly target YAP1. **A** Putative complementary sites within miR-200a-3p andYAP1 predicted by bioinformatics analysis (TargetScan). **B** Dual luciferase reporter assays demonstrated that YAP1 is a direct target of miR-200a-3p. **C-D** YAP1 had high expression in BC tissues compared with adjacent normal tissues. **E** YAP1 had high expression in BC cell lines. **F** Positive correlations between the expression of hsa_circ_0005273 and YAP1 were found with Pearson’s correlation analysis in BC tissue samples (n = 120). **G** Negative correlations between the expression of miR-200a-3p and YAP1 were found with Pearson’s correlation analysis in BC tissue samples (n = 120). **H** The protein levels of YAP1/p-YAP1 to be increased after transfection of miR-200a-3p inhibitor in BC cells by Western blotting. **I, K, M and O** Si-YAP1 rescued the promotive effects of miR-200a-3p-inhibitor in BC cells by MTT assay and transwell assays. **J, L and N** Western blotting showed that si-YAP1 could partly rescue the high expression of YAP1, p-YAP1 and PCNA caused by miR-200a-3p-inhibitor in BC cells. *p < 0.05, **p < 0.01,*** p < 0.001,**** p < 0.0001

In accordance with the prediction of TargetScan (http://www.targetscan.org/vert_71/) , YAP1 was found to be the potential target of miR-200a-3p(Fig. 5a). By constructing plasmid and mutant vectors containing 3′-UTRs with wild-type and mutant sequences, a dual-fluorescein reporter assay confirmed that YAP1 was the direct target of miR-200a-3p(Fig. 5b). The expression levels of YAP1 were tested in both BC tissues and cell lines through RT-qPCR and the results showed high expression levels of YAP1 in BC (Fig. 5c-e). Furthermore, the negative relevance between miR-200a-3p and YAP1 as well as the positive relevance between hsa_circ_0005273 and YAP1 were analyzed with Pearson’s correlation analysis (Fig. 5f,g). Western blotting was performed to verify the effect of miR-200a-3p on YAP1. As expected, the protein levels of YAP1 and p-YAP1 increased when transfected with miR-200a-3p-inhibitor (Fig. 5h). Then, rescue assays were designed in BC cells. As shown in Fig. 5, BC cells transfected with miR-200a-3p-inhibitor were co-transfected with a specific siRNA of YAP1(si-YAP1). The results of MTT and transwell assays indicated that introduction of si-YAP1 could decreased BC cell proliferation and migration induced by miR-200a-3p-inhibitor (Fig. 5i,k,m,o). Meanwhile, elevating effect of miR-200a-3p-inhibitor on YAP1/p-YAP1 protein levels were reversed by si-YAP1(Fig. 5j,l,n).

### Hsa_circ_0005273 upregulated YAP1 through targeting miR-200a-3p

**Fig. 6:**
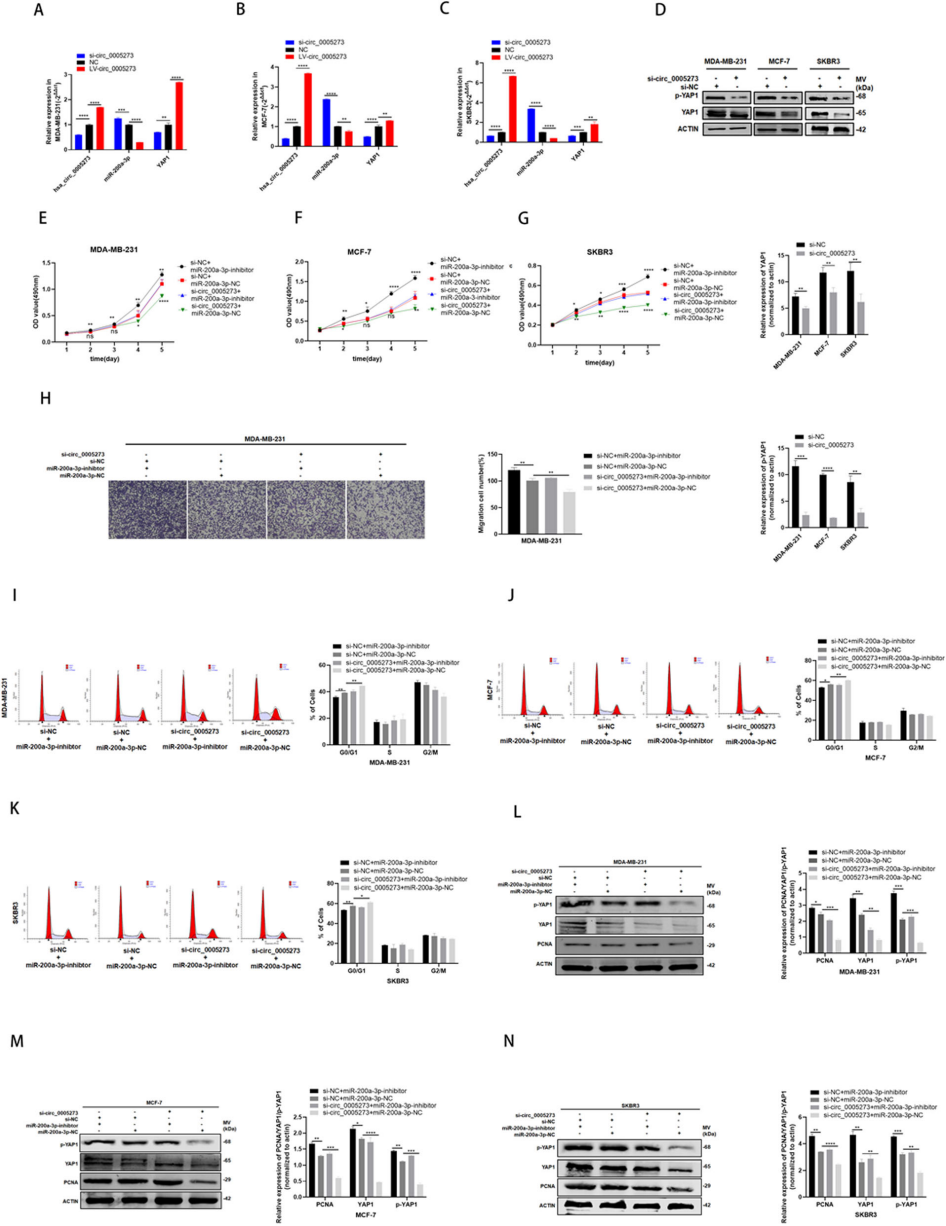
Hsa_circ_0005273 upregulated YAP1 through targeting miR-200a-3p. **A-C** the expression of miR-200a-3p increased and the expression of YAP1 decreased after hsa_circ_0005273 was downregulated by RT-qPCR in BC cells. **D** The protein levels of YAP1/p-YAP1 to be decreased after transfection of si-circ_0005273 in BC cells by Western blotting. **E-H** MiR-200a-3p-inhibitor rescued the promotive effects of hsa_circ_0005273 in BC cells by MTT assay and transwell assays. **I-K** MiR-200a-3p-inhibitor reversed cell cycle arrest induced by si-hsa_circ_0005273. **L-N** Western blotting showed that miR-200a-3p-inhibitor can partly rescue the low expression of YAP1, p-YAP1 and PCNA caused by si-circ_0005273 in BC cells. *p < 0.05, **p < 0.01,*** p < 0.001,**** p < 0.0001

Considering the interaction between hsa_circ_0005273 and miR-200a-3p as well as miR-200a-3p and YAP1, we wanted to make a thorough inquiry about whether hsa_circ_0005273 regulates the expression of YAP1. Firstly, RT-qPCR was used to detect the expression of miR-200a-3p and YAP1 in MDA-MB-231, MCF-7 and SKBR3 cells respectively infected with si-circ_0005273 or LV-circ_0005273(Fig. 6a-c). Results of RT-qPCR indicated that the expression of miR-200a-3p increased and the expression of YAP1 decreased after downregulating hsa_circ_0005273 expression. Consistently, the expression of miR-200a-3p decreased and the expression of YAP1 increased after upregulating hsa_circ_0005273 expression. Secondly, the results of western blotting revealed that the protein levels of YAP1 and p-YAP1 decreased when transfected with si-circ_0005273 (Fig. 6d). To determine whether hsa_circ_0005273 affects YAP1 via miR-200a-3p and the biological function of hsa_circ_0005273-miR-200a-3p-YAP1 axis in BC progression, we designed rescue assays. We transfected a combination of both si-circ_0005273 and miR-200a-3p inhibitor to further detect the expression of YAP1 and changes of BC cells. According to the results of rescue assays, downregulating hsa_circ_0005273 induced cell proliferation and migration reduction as well as cell cycle arrest could be rescued by miR-200a-3p inhibitor (Fig. 6e-k). Moreover, according to the results of western blotting, downregulating hsa_circ_0005273 induced YAP1 and p-YAP1 expression reduction could be recovered by miR-200a-3p inhibitor (Fig. 6l-n). Based on the results above, we confirmed that hsa_circ_0005273 acted as an oncogene in BC cells via miR-200a-3p/YAP1 axis.

### Hsa_circ_0005273 inactivated hippo signaling pathway

**Fig. 7:**
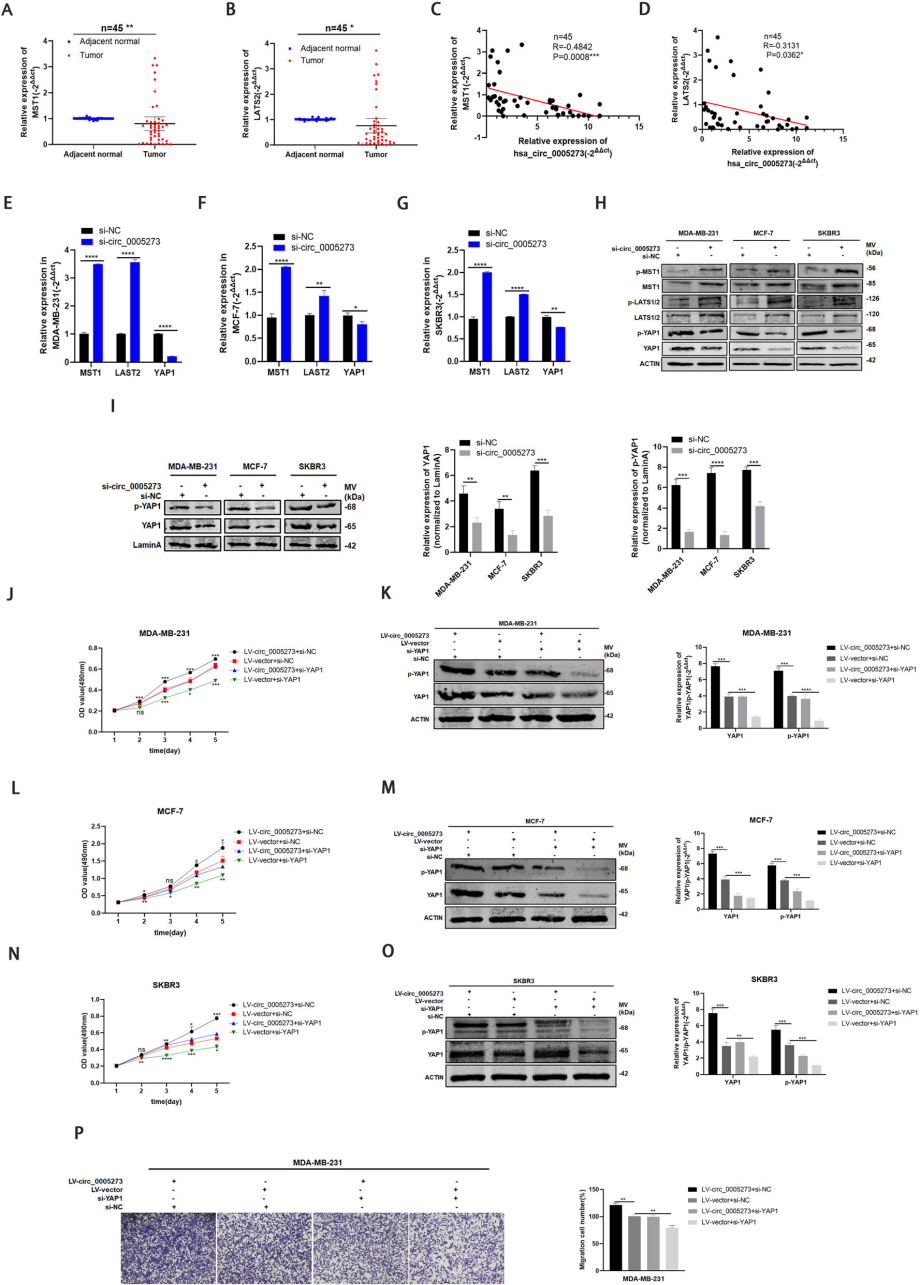
Hsa_circ_0005273 inactivated Hippo signaling pathway. **A-B** LATS2/MST1 had low expression in BC tissues compared with adjacent normal tissues. **C-D** Negative correlations between the expression of hsa_circ_0005273 and LATS2/MST1 were found with Pearson’s correlation analysis in BC tissue samples (*n* = 45). **E-G** MRNA levels of MST1, LATS2 and YAP1 were tested in BC cells transfected with si-circ_0005273. **H** Protein levels of MST1, LATS1/2, YAP1 and their phosphorylation status were tested in BC cells transfected with si-circ_0005273. **I** Protein levels of YAP1 and p-YAP1 were tested in nucleus of si-circ_0005273 transfected BC cells. **J, L, N and P** Si-YAP1 rescued the promotive effects of hsa_circ_0005273 in BC cells by MTT assay and transwell assays. **K, M and O** Western blotting showed that si-YAP1 can partly rescue the high expression of YAP1 and p-YAP1 caused by LV-circ_0005273 in BC cells. *p < 0.05, **p < 0.01,*** p < 0.001,**** p < 0.0001

The dysregulation and inactivation of the Hippo pathway always results in cancer initiation and progression [14]. Previous studies have reported that inactivation of Hippo pathway resulted in downregulation of LATS1/2 and MST1/2 as well as upregulation of YAP1. To investigate whether hsa_circ_0005273 could inactivate Hippo signaling pathway, we further detect the expression of LATS1/2 and MST1 in BC. The results of RT-qPCR showed that LATS2 and MST1 was low-expressed in BC tissues, and there was a negative correlation between LATS2/MST1 and hsa_circ_0005273 (Fig. 7a-d). Furthermore, protein and mRNA levels of LATS1/2 and MST1 test in si-circ_0005273 transfected MDA-MB-231, MCF-7 and SKBR3 cells, showed that LATS1/2, MST1 and their phosphorylation status were upregulated while YAP1/p-YAP1 were downregulated (Fig. 7e-h). The nuclear translocation of YAP1 is also essential for the activation of hippo signaling pathway [18]. We further investigate the expression levels of YAP1 between the nucleus and cytoplasm. Consistently, protein levels of YAP1 and p-YAP1 decreased in nucleus of si-circ_0005273 transfected BC cells while increased in miR-200a-3p-inhibtor transfected BC cells (Fig. 7i, S1G). In addition, we suppressed the YAP-1 expression in hsa_circ_0005273-overexpressing BC cells. The results of MTT assays and transwell assays showed that upregulating hsa_circ_0005273 induced BC cells tumorigenesis could be rescued by si-YAP1, consistently with the results of western blotting (Fig. 7j-p). Taking all results above, we confirmed that upregulated hsa_circ_0005273 inactivated Hippo pathway in BC.

### Hsa_circ_0005273 promotes BC tumor growth in vivo

**Fig. 8:**
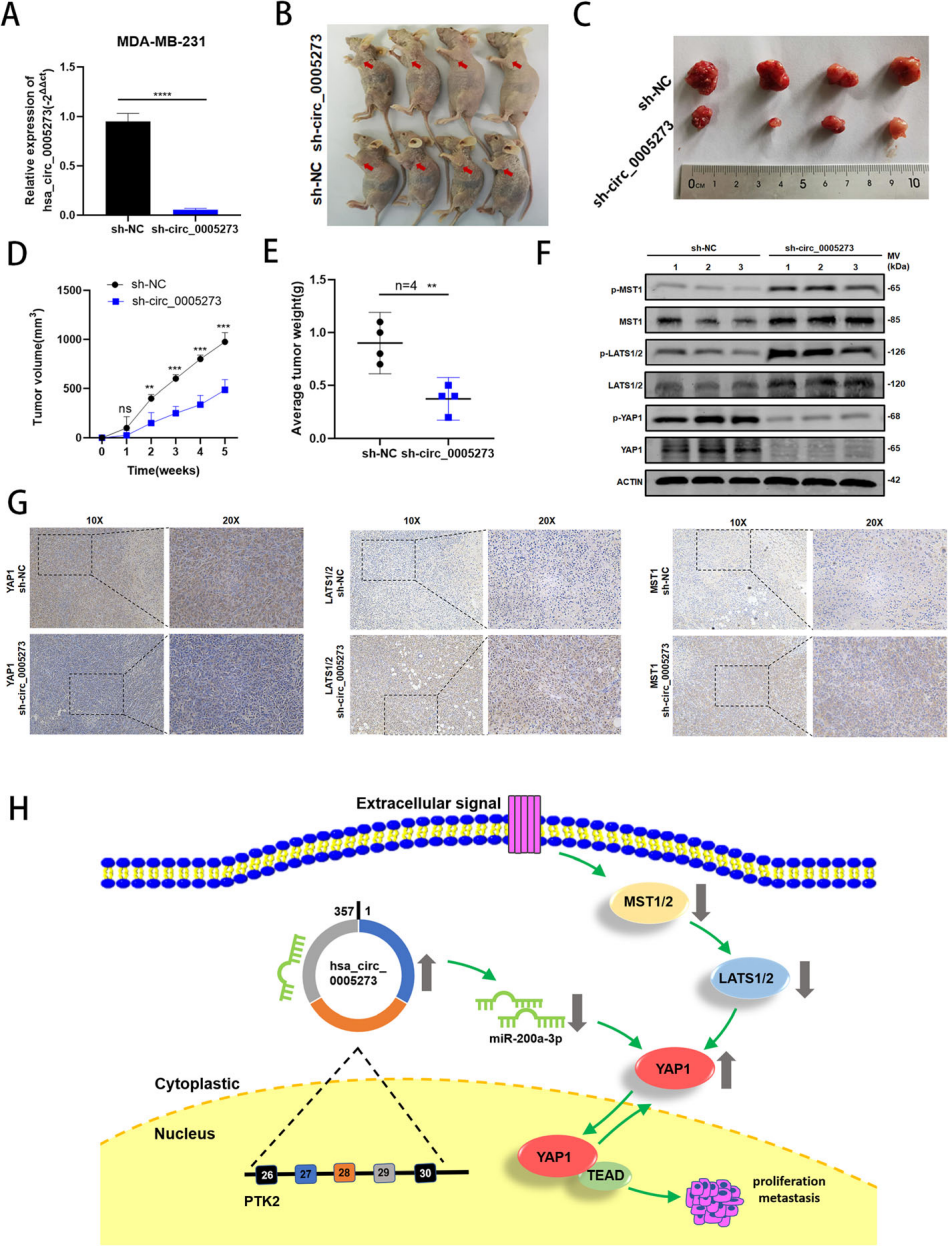
Hsa_circ_0005273 promotes BC tumor growth in vivo. **A** Hsa_circ_0005273 knockdown efficiency using shRNAs in MDA-MB-231 cells was verified by RT-qPCR. **B** Representative images of nude mice injected with MDA-MB-231 cells (4 mice per group). **C** Representative images of xenograft tumors in nude mice. **D** The growth curves of xenografts. **E** Average tumor weight of nude mice. **F** Extract protein from tumors and measuring the expression of YAP1, MST1, LAST1/2 and their phosphorylation status by Western blotting. **G** Immunohistochemistry (IHC) staining of YAP1, MST1 and LATS1/2 in xenografts. **H** The mechanism diagram was generated to illustrate the mechanism of hsa_circ_0005273-miR-200a-3p-YAP1-Hippo pathway in BC. *p < 0.05, **p < 0.01,*** p < 0.001,**** p < 0.0001

To further examine the role of hsa_circ_0005273 in BC tumorigenesis, we carried out the xenograft tumor assay in MDA-MB-231 cells stably infected by lentiviral, which were transfected with lv-sh_circ_0005273 or lv-sh_NC(Fig. 8a). Then, the cells were injected into nude mice (Fig. 8b). The mice tumors were photographed, measured and weighted, indicating that knockdown hsa_circ_0005273 expression could markedly decrease the tumor volume and weight compared with the negative controls (Fig. 8c-e). The results of xenograft tumor assay further supported the oncogenic role of hsa_circ_0005273 in BC tumorigenesis. Moreover, the xenograft tumors protein was extracted and the expression of YAP1, MST1, LATS1/2 and their phosphorylation status were detected by western blotting and IHC (Fig. 8f,g). The results confirmed that the expression of YAP1 decreased and the expression of LATS1/2, MST1 increased in the lower hsa_circ_0005273 expression group. Taking all results in vivo and in vitro together, we confirmed that hsa_circ_0005273 inactivated Hippo pathway in BC. The mechanism of hsa_circ_0005273-miR-200a-3p-YAP1-Hippo pathway in BC was generated in Fig. 8h.

## Discussion

The past few decades have witnessed the rapid research progress of circRNAs and a number of circRNAs have been confirmed to participate in the tumorigenesis of multiple cancers, especially in BC. CircRNAs have covalently closed ring structure with no 5′ caps and 3′ ploy-A tails, thus they are stable and insensitive to RNase R [19]. Considerable quantities of circRNAs are located in the cytoplasm and few are found in the nucleus [20]. Considering the stability, long half-life period of circRNAs as well as tissue-specific expression, circRNAs have distinct advantages to act as potential biomarkers of cancer diagnosis and prognosis [21].

Through analyzing GEO database and our experiment results, we identified a novel circRNA, hsa_circ_0005273, was remarkably upregulated in BC tissues. More importantly, high expression hsa_circ_0005273 was positively associated with tumor size, TNM stage, lymph node metastasis and distant metastasis in BC patients. Knocking down hsa_circ_0005273 could inhibit the proliferation, migration and cell cycle of BC cells as well as the growth of tumors in vivo, indicating its promotive role in BC tumorigenesis.

Previous studies have demonstrated various physiological functions of circRNAs, such as circRNAs serving as competing endogenous RNAs (ceRNAs) or natural miRNA sponges [22, 23], interacting with proteins [24–26], regulating gene transcription [20, 27] and translation [28–31]. Normally, the function of circRNAs are related to their subcellular localization. CircRNAs enriched in cytoplasm contain a large number of miRNA response elements MER (miRNA response element), thus allows them to act as miRNA sponges and reverse the inhibitory effect of miRNA on its target genes [3, 32]. Through FISH assay and subcellular fractionation, we confirmed that hsa_circ_0005273 was mainly located in cytoplasm of BC cells. Therefore, we effort to explore the molecular mechanism of hsa_circ_0005273 as a miRNA sponge. RIP assay was first performed to verify that hsa_circ_0005273 could target miRNAs in an AGO2 manner. Then, dual luciferase reporter assays, RNA pull-down analysis and FISH study were conducted to demonstrated the interaction between hsa_circ_0005273, miR-200a-3p and YAP1. Additionally, we observed YAP1 was positively correlated with hsa_circ_0005273 expression in both mRNA and protein levels. Eventually, rescue assays further verified hsa_circ_0005273 could regulate YAP1 via targeting miR-200a-3p.

As a tumor suppressor pathway, inactivation of Hippo signaling pathway could culminate epithelial-to-mesenchymal transition (EMT), cancer stem cell generation and therapeutic resistance [18]. YAP1, primarily as the downstream gene of Hippo signaling pathway, was demonstrated as an important oncogene in a considerable number of cancer types. Normally, upstream factors of the Hippo pathway, such as MST1/2 and LATS1/2, operate an inhibitory phosphorylation of YAP1, thereby inhibiting it nuclear translocation and YAP-mediated transcription [33, 34]. When the Hippo signaling pathway is inactivated, YAP1 translocate to the nucleus where they interact with TEADs, inducing target gene expression [35–37]. Considering YAP1 as a critical factor in Hippo pathway, we hypothesized that hsa_circ_0005273 could inactivate Hippo signaling pathway via targeting YAP1. Thereafter, we verified that LATS1/2 and MST1expression were negative related to hsa_circ_0005273. In addition, the protein and mRNA levels of LATS1/2, MST1 and their phosphorylation status were negatively regulated by hsa_circ_0005273. More importantly, hsa_circ_0005273 promoted YAP1 translocate to the nucleus, which is a key evidence of Hippo pathway inactivation [18].

This study is first to suggest that the hsa_circ_0005273/miR-200a-3p/YAP1/Hippo pathway may play a key role in the progression of BC and provide a novel strategy for inhibiting YAP1.

## Conclusion

In summary, we identified that hsa_circ_0005273 is highly expressed and acts as an oncogene in BC. Moreover, hsa_circ_0005273 could regulate YAPl via serving as a sponge of miR-200a-3p, thereby inactivating Hippo signaling pathway. The hsa_circ_0005273/miR-200a-3p/YAP1/ Hippo pathway axis may provide a potential novel biomarker and therapeutic target for BC.

## Supplementary Information

**Additional file 1 Fig. S1 A** Relative expression of hsa_circ_0005273 in GSE113230. **B** Wound healing assays were performed in MCF-7 and SKBR3 treated with si-circ_0005273. **C** The mRNA level of PTK2 had no changes after hsa_circ_0005273 was downregulated by RT-qPCR in BC cells. **D** The protein level of PTK2 had no changes after hsa_circ_0005273 was downregulated by Western blotting in BC cells. **E** Protein levels of YAP1 and p-YAP1 were tested in nucleus of miR-200a-3p-inhibitor transfected BC cells. Student’s t-test, *p < 0.05, **p < 0.01,*** *p* < 0.001,**** *p* < 0.0001.

**Additional file 2 Table S1** Primers and siRNAs used in this study.

**Additional file 3 Table S2** The relationship between the expression of hsa_circ_0005273 and various clinicopathological variables of basal-like cohort in BC patients.

**Additional file 4 Table S3** The relationship between the expression of hsa_circ_0005273 and various clinicopathological variables of Her-2-like cohort in BC patients.

**Additional file 5 Table S4** The relationship between the expression of hsa_circ_0005273 and various clinicopathological variables of luminal-like cohort in BC patients.

**Additional file 6.** Original western blotting images in this study.

## Data Availability

The datasets used and analyzed during the current study are available from the corresponding author on reasonable request.

## Abbreviations

circRNA: Circular RNA
ncRNA: Non-coding RNA
GEO: Gene Expression Omnibus
PTK2: Protein Tyrosine Kinase 2
YAP1: yes-associated protein
LATS1/2: Large tumor suppressor 1/2
MST1/2: Mammalian Ste20-like kinases 1/2
GAPDH: Glyceraldehyde-3-Phosphate Dehydrogenase
RT-qPCR: Quantitative real-time polymerase chain reaction
