# Correction to: Hsa_circ_0005273 facilitates breast cancer tumorigenesis by regulating YAP1-hippo signaling pathway

**DOI:** 10.1186/s13046-021-02160-w

**Published:** 2021-11-16

**Authors:** Xuehui Wang, Changle Ji, Jiashu Hu, Xiaochong Deng, Wenfang Zheng, Yunhe Yu, Kaiyao Hua, Xiqian Zhou, Lin Fang

**Affiliations:** 1 Department of Thyroid and Breast Surgery, Shanghai Tenth People’s Hospital, School of Medicine, Tongji University, Shanghai, 200072 China; 2 Nanjing Medical University, Nanjing, 211166 China

**Correction to: J Exp Clin Cancer Res 40, 29 (2021)**

**https://doi.org/10.1186/s13046-021-01830-z**

Following publication of the original article [1], the authors identified minor errors in Figs. 2, 3 and 4, specifically:

- Figs. 2I, 2T, 4 H and 4 I: mismatched images were introduced in the colony formation assays
- Fig. 3D: the statistical data of Fig. 3C was mistakenly duplicated in Fig. 3D

**Fig. 2:**
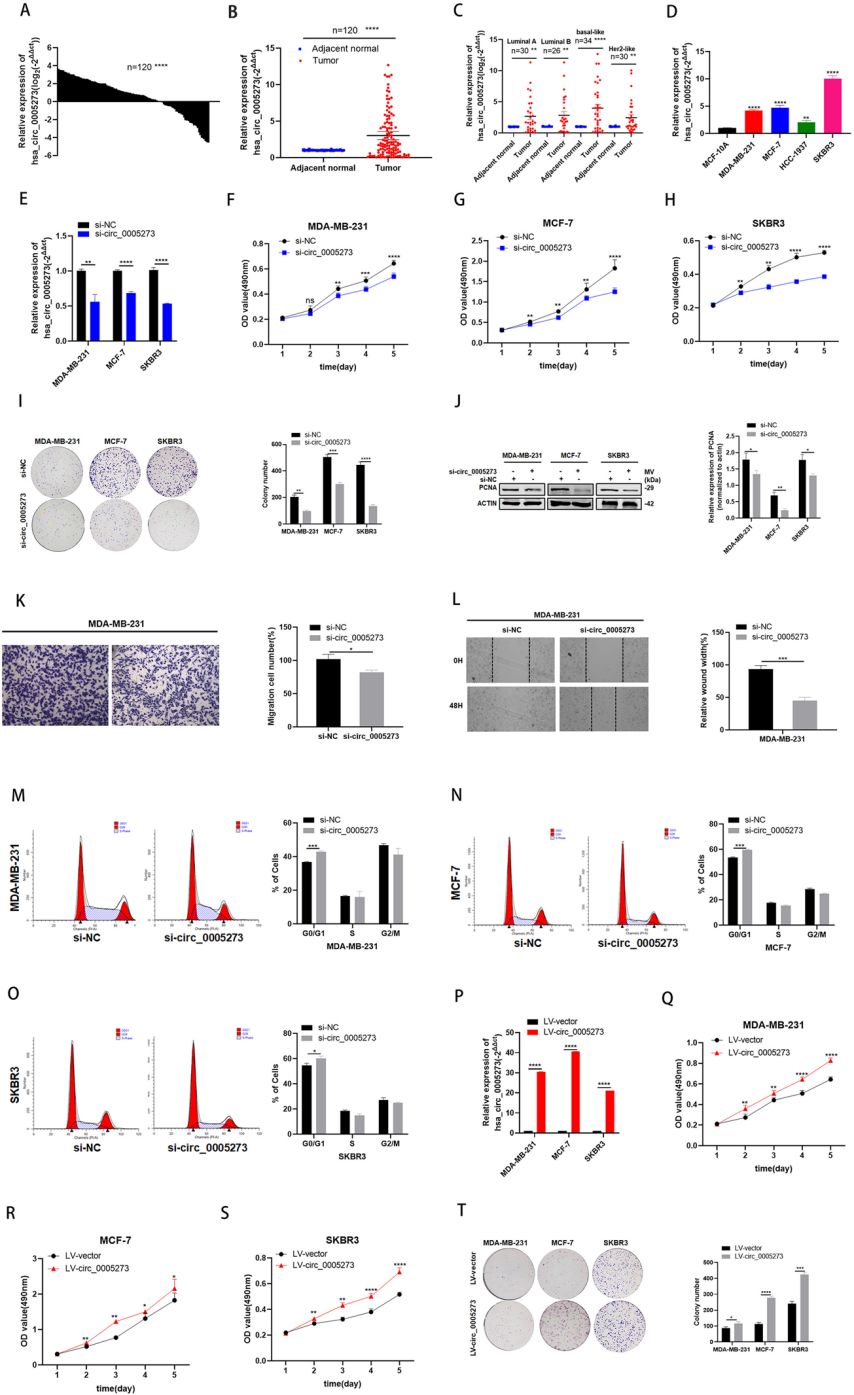
Hsa_circ_0005273 is highly expressed in BC and exerts oncogenic effects in BC cells. **A-B** Hsa_circ_0005273 was highly expressed in tumor tissues compared with adjacent normal tissues. **C** Expression of hsa_circ_0005273 in basal-like cohort, Her2-like cohort, luminal-A and luminal-B cohort, respectively. **D** Relative expression of hsa_circ_0005273 in BC cell lines. **E** Expression of hsa_circ_0005273 was confirmed by RT-qPCR in BC cells transfected with si-NC or si-circ_0005273. **F-H** Effect of si-circ_0005273 on proliferation in BC cells by MTT assay. **I** Effect of si-circ_0005273 on proliferation in BC cels by colony formation assay. **J** Effect of si-circ_0005273 on proliferation in BC cells by western blotting. **K** Cell migration assays were performed in MDA-MB-231 using transwell chambers. **L** Wound healing assays were performed in MDA-MB-231 treated with sicirc_0005273. **M-O** Cell cycle assays were performed in BC cells treated with si-circ_0005273. **P** Expression of hsa_circ_0005273 was confirmed by RT-qPCR in BC cells transfected with LV-vtctor or LV-circ_0005273. **Q-S** Effect of LV-circ_0005273 on proliferation in BC cell lines by MTT assay. **T** Effect of LV-circ_0005273 on proliferation in BC cell lines by colony formation assay. *p < 0.05, **p < 0.01,*** *p* < 0.001,**** *p* < 0.0001

**Fig. 3:**
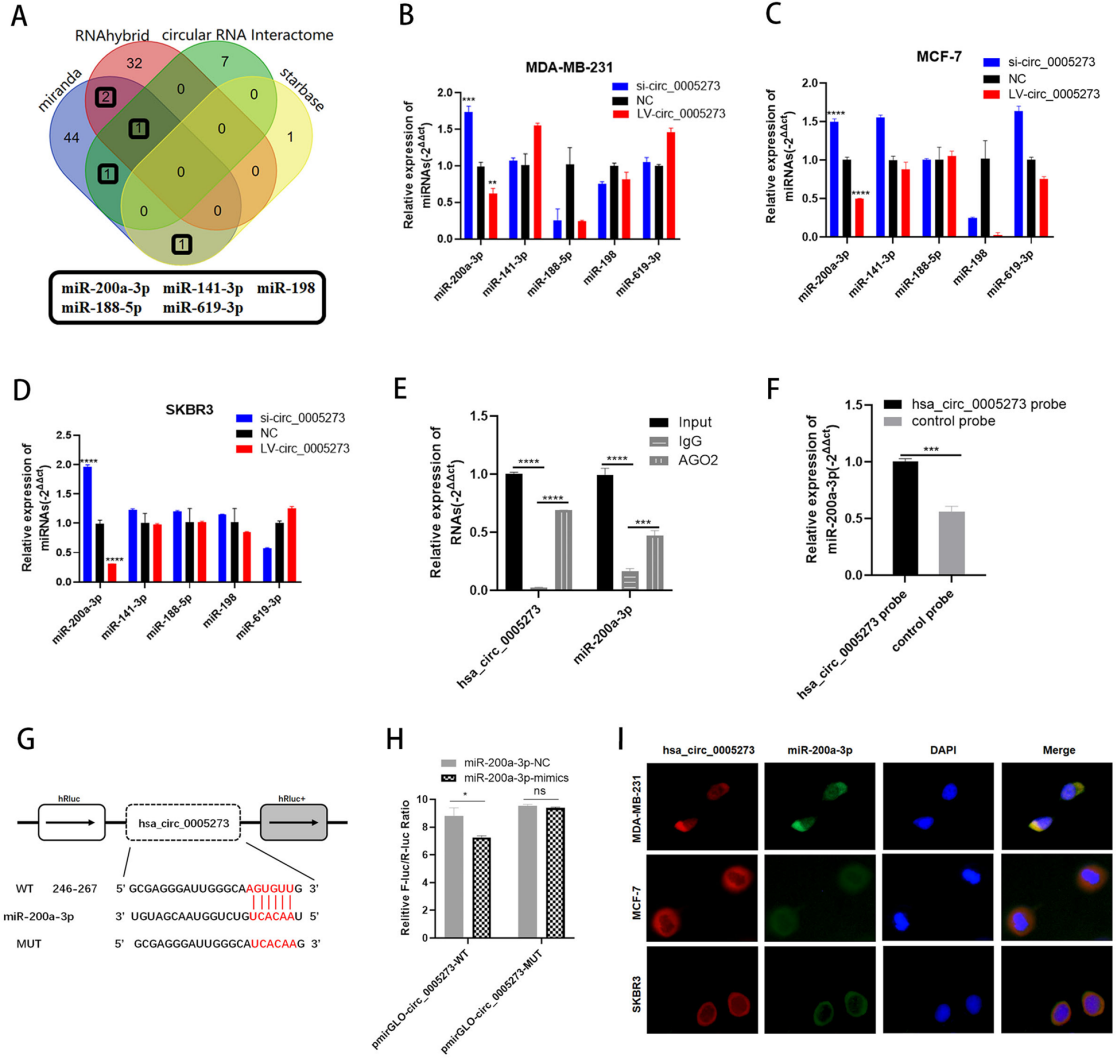
Hsa_circ_0005273 serves as a sponge for miR-200a-3p. **A** Venn diagram showing the potential target miRNAs of hsa_circ_0005273. **B-D** Expression of miRNAs in BC cells transfected with si-circ_0005273 or LV-circ_0005273. **E** RIP experiments were performed in HEK293T cells, and the co-precipitated RNA was subjected to RT-qPCR for hsa_circ_0005273 and miR-200a-3p. **F** MiR-200a-3p was pulled down and enriched with hsa_circ_0005273 specific probe and then detected by RT-qPCR. **G** Putative complementary sites within miR-200a-3p and hsa_circ_0005273 predicted by bioinformatics analysis (starbase). **H** Dual luciferase reporter assays demonstrated that miR-200a-3p is a direct target of hsa_circ_0005273. **I** Detection of colocalization of hsa_circ_0005273 and miR-200a-3p in cytoplasm by RNA FISH assay (magnification, × 400). Green, miR-200a-3p; Red, hsa_circ_0005273; Blue, DAPI. *p < 0.05, **p < 0.01,*** *p* < 0.001,**** *p* < 0.0001

**Fig. 4:**
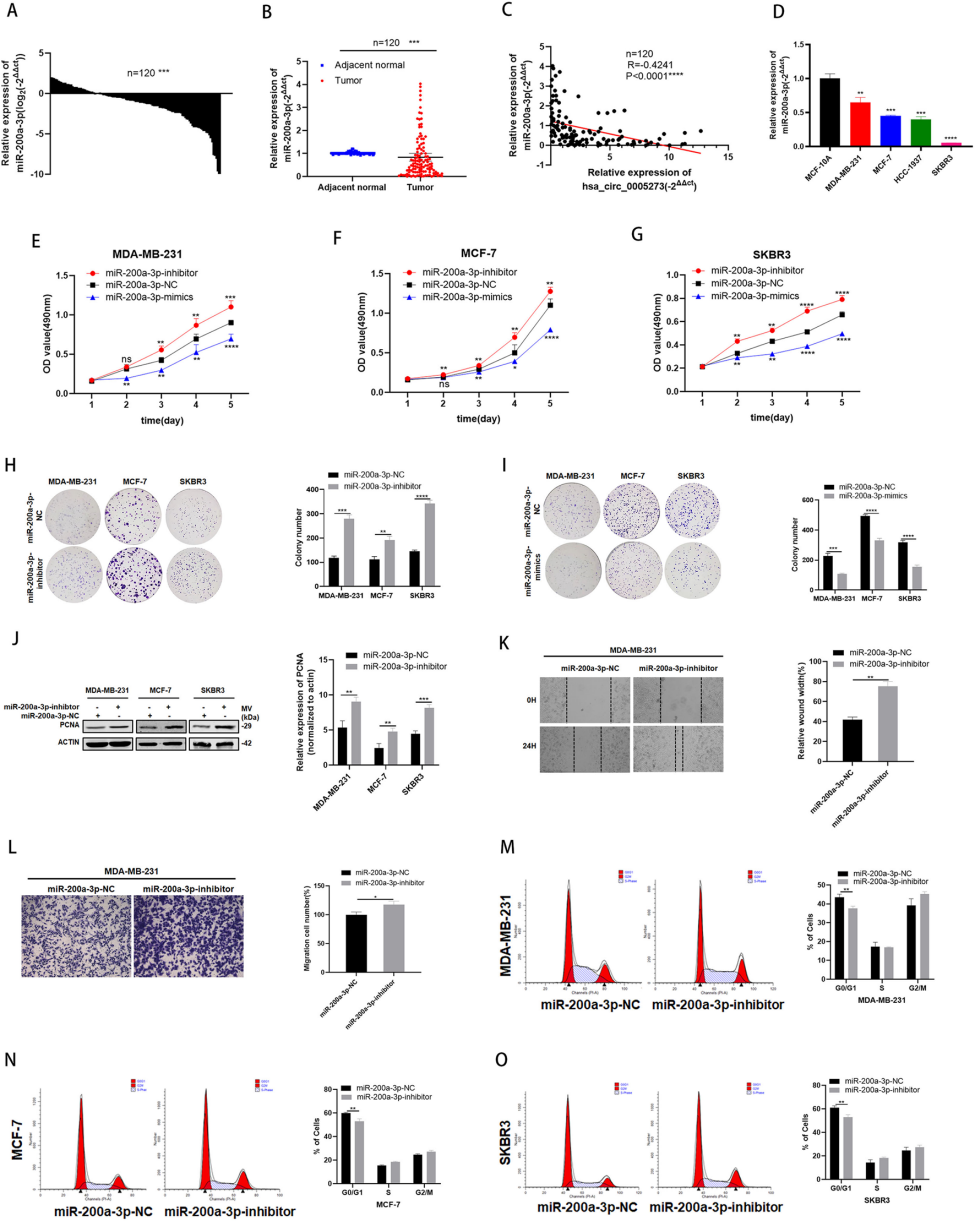
MiR-200a-3p is expressed at low levels and acts as a tumor suppressor in BC cells. **A-B** MiR-200a-3p had low expression in BC tissues compared with adjacent normal tissues. **C** Correlations between the expression of hsa_circ_0005273 and miR-200a-3p were found with Pearson’s correlation analysis in BC tissue samples (*n* = 120). **D** MiR-200a-3p had low expression in BC cell lines. **E-G** Effect of miR-200a-3p-mimics on proliferation in BC cells by MTT formation assay. **H-I** Effect of miR-200a-3p-inhibitor on proliferation in BC cells by colony formation assay. **J** Effect of miR-200a-3p-inhibitor on proliferation in BC cells by western blotting. **K** Wound healing assays were performed in MDA-MB-231 cell treated with miR-200a-3p-inhibitor. **L** Cell migration assays were performed in MDA-MB-231 cells using Transwell chambers. **M-O** Cell cycle assays were performed in BC cells treated with miR-200a-3p-inhibitor. *p < 0.05, **p < 0.01,*** *p* < 0.001,**** *p* < 0.0001

The authors provided the Journal with the original data files. The corrected figures are provided here. The correction does not have any effect on the results or conclusions of the paper. The original article has been corrected.
