# Supplementary figures and images for: Hsa_circ_0005273 facilitates breast cancer tumorigenesis by regulating YAP1-hippo signaling pathway
Source: J Exp Clin Cancer Res. 2021 Jan 12;40:29. doi: 10.1186/s13046-021-01830-z (PMC7802350; doi:10.1186/s13046-021-01830-z)

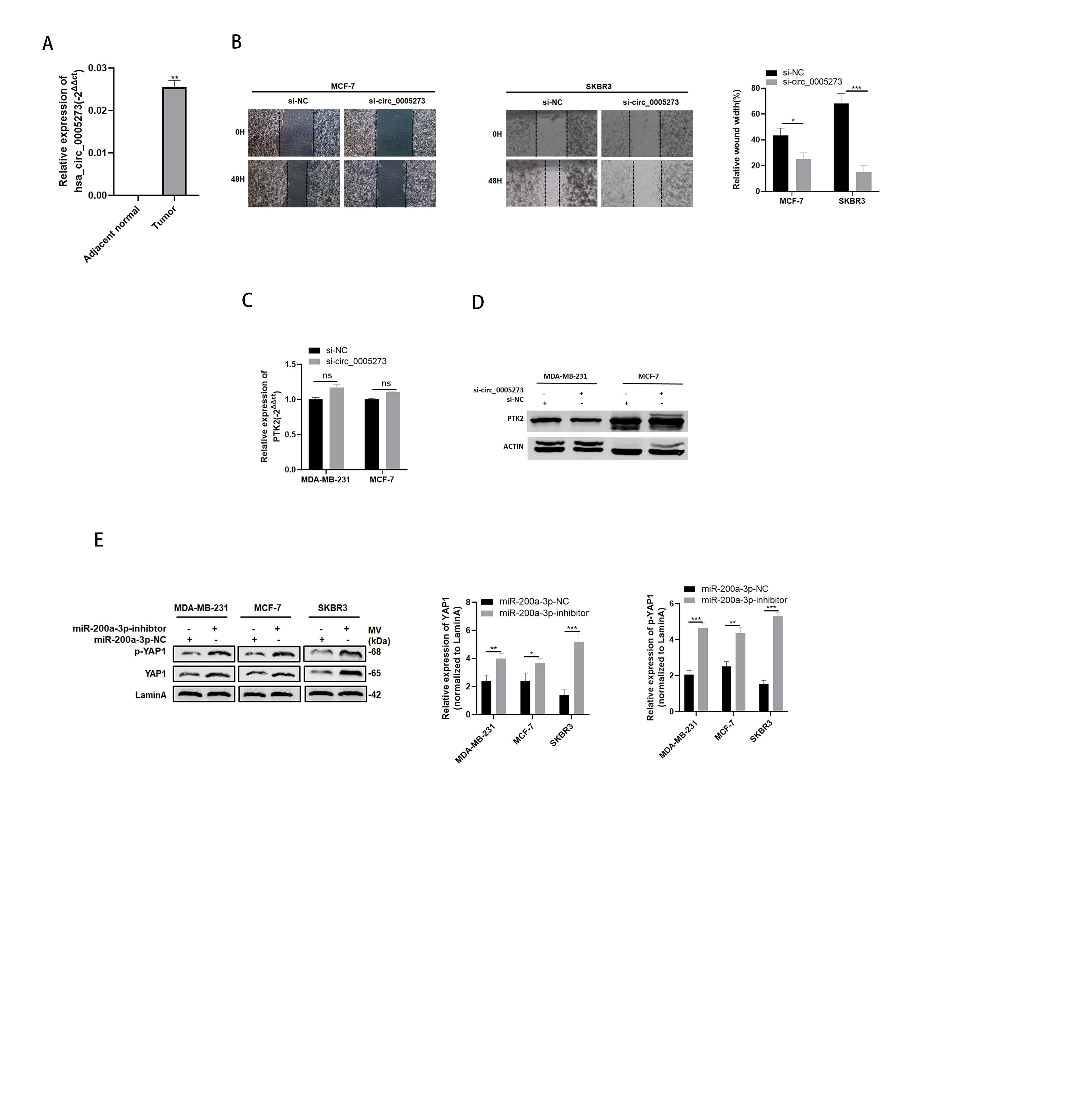

Supplement: Supplementary file 1 — Additional file 1 Fig. S1 A Relative expression of hsa_circ_0005273 in GSE113230. B Wound healing assays were performed in MCF-7 and SKBR3 treated with si-circ_0005273. C The mRNA level of PTK2 had no changes after hsa_circ_0005273 was downregulated by RT-qPCR in BC cells. D The protein level of PTK2 had no changes after hsa_circ_0005273 was downregulated by Western blotting in BC cells. E Protein levels of YAP1 and p-YAP1 were tested in nucleus of miR-200a-3p-inhibitor transfected BC cells. Student’s t-test, *p < 0.05, **p < 0.01,*** p < 0.001,**** p < 0.0001. [file 13046_2021_1830_MOESM1_ESM.jpg]
