# Supplementary material for: Hsa_circ_0005273 facilitates breast cancer tumorigenesis by regulating YAP1-hippo signaling pathway
Source: J Exp Clin Cancer Res. 2021 Jan 12;40:29. doi: 10.1186/s13046-021-01830-z (PMC7802350; doi:10.1186/s13046-021-01830-z)
Supplement: Supplementary file 2 — Additional file 2 Table S1 Primers and siRNAs used in this study. [file 13046_2021_1830_MOESM2_ESM.docx]

**Table S1 Primers and siRNAs used in this study**

| **Gene** | **Sequences** |
| --- | --- |
| 18S-F  18S-R | 5'-TAGAGGGACAAGTGGCGTTC-3'  5'-CGCTGAGCCAGTCAGTGT-3' |
| hsa_circ_0005273-F | 5′-AAAAAGAGGAAAGATTTCTGCCC-3′ |
| hsa_circ_0005273-R  GAPDH-F  GAPDH-R | 5′- TTTGTACCATGTGCTGTGGGC-3′  5'-CAGGAGGCATTGCTGATGAT-3'  5'-GAAGGCTGGGGCTCATTT-3' |
| PTK2-F | 5′-TGGGCGGAAAGAAATCCTGC-3′ |
| PTK2-R | 5′-GGCTTGACACCCTCGTTGTA-3’ |
| YAP1-F | 5’-GCGUAGCCAGUUACCAACATT-3' |
| YAP1-R | 5'-UGUUGGUAACUGGCUACGCTT-3' |
| LATS2-F | 5’-CGCCTGTGTTCCAGGCTGTGG-3’ |
| LATS2-R | 5’-CCAGGTGCTGCTGTTTGGGAG-3’ |
| MST1-F | 5’-TCTAGCCATGGCTCACTAT-3’ |
| MST1-R | 5’-ACACTGGTCTAACGTGGAT-3’ |
| miR-200a-3p-F | 5’-GGCTAACACTGTCTGGTAA CGATG-3’ |
| miR-200a-3p-R | 5’ -GTGCAGGGTCCGAGGT-3’ |
| U6-F | 5'-CAAATTCGTGAAGCGTTCCATAT-3' |
| U6-R | 5'-GCTTCACGAATTTGCGTGTCATCCTTGC-3' |
| si-circ_0005273- Sense | 5’-AGGAAAGAUUUCUGCCCAGCATT-3’ |
| si-circ_0005273- Anti-sense | 5’-UGCUGGGCAGAAAUCUUUCCUTT-3’ |
| si-NC- Sense | 5’-UUCUCCGAACGUGUCACGUTT-3’ |
| si-NC- Anti-sense | 5’-ACGUGACACGUUCGGAGAATT-3’ |
