# Supplementary material for: Hsa_circ_0005273 facilitates breast cancer tumorigenesis by regulating YAP1-hippo signaling pathway
Source: J Exp Clin Cancer Res. 2021 Jan 12;40:29. doi: 10.1186/s13046-021-01830-z (PMC7802350; doi:10.1186/s13046-021-01830-z)
Supplement: Supplementary file 3 — Additional file 3 Table S2 The relationship between the expression of hsa_circ_0005273 and various clinicopathological variables of basal-like cohort in BC patients. [file 13046_2021_1830_MOESM3_ESM.docx]

| Patients Characteristics | Total | hsa_circ_0005273 expression | |  |
| --- | --- | --- | --- | --- |
|  |  | High (*N=24)* | Low (*N=10)* | P value* |
| Age |  |  |  | 0.1500 |
| <60 | 14 | 8 | 6 |  |
| ≥60 | 20 | 16 | 4 |  |
| TNM stage |  |  |  | 0.0836 |
| Ⅰ and Ⅱ | 16 | 9 | 7 |  |
| Ⅲ and Ⅳ | 18 | 15 | 3 |  |
| Tumor size(cm) |  |  |  | 0.0517 |
| ≤2 | 12 | 6 | 6 |  |
| ＞2 | 22 | 18 | 4 |  |
| Lymph node metastasis |  |  |  | 0.0130* |
| negative | 16 | 8 | 8 |  |
| positive | 18 | 16 | 2 |  |
| Distant metastasis |  |  |  | 0.0239* |
| No | 25 | 15 | 10 |  |
| Yes | 9 | 9 | 0 |  |

**Table S2** **The relationship between the expression of hsa_circ_0005273 and various clinicopathological variables of basal-like cohort in BC patients.**

* p < 0.05

**Table S2** The relationship between the expression of hsa_circ_0005273 and various clinicopathological variables of basal-like cohort in BC patients (n=34). High expression of hsa_circ_0005273 was positively associated with lymph node metastasis and distant metastasis, but had no correlation with age, TNM stage and tumor size.
