# Supplementary material for: Hsa_circ_0005273 facilitates breast cancer tumorigenesis by regulating YAP1-hippo signaling pathway
Source: J Exp Clin Cancer Res. 2021 Jan 12;40:29. doi: 10.1186/s13046-021-01830-z (PMC7802350; doi:10.1186/s13046-021-01830-z)
Supplement: Supplementary file 4 — Additional file 4 Table S3 The relationship between the expression of hsa_circ_0005273 and various clinicopathological variables of Her-2-like cohort in BC patients. [file 13046_2021_1830_MOESM4_ESM.docx]

| Patients Characteristics | Total | hsa_circ_0005273 expression | |  |
| --- | --- | --- | --- | --- |
|  |  | High (*N=22)* | Low (*N=8)* | P value* |
| Age |  |  |  | 0.4456 |
| <60 | 14 | 10 | 4 |  |
| ≥60 | 16 | 12 | 4 |  |
| TNM stage |  |  |  | 0.0183* |
| Ⅰ and Ⅱ | 12 | 6 | 6 |  |
| Ⅲ and Ⅳ | 18 | 16 | 2 |  |
| Tumor size(cm) |  |  |  | 0.0183* |
| ≤2 | 12 | 6 | 6 |  |
| ＞2 | 18 | 16 | 2 |  |
| Lymph node metastasis |  |  |  | 0.4239 |
| negative | 19 | 13 | 6 |  |
| positive | 11 | 9 | 2 |  |
| Distant metastasis |  |  |  | 0.1951 |
| No | 26 | 18 | 8 |  |
| Yes | 4 | 4 | 0 |  |

**Table S3** **The relationship between the expression of hsa_circ_0005273 and various clinicopathological variables of Her-2-like cohort in BC patients.**

* p < 0.05

**Table S3** The relationship between the expression of hsa_circ_0005273 and various clinicopathological variables of Her-2-like cohort in BC patients (n=30). High expression of hsa_circ_0005273 was positively associated with TNM stage and tumor size, but had no correlation with age, lymph node metastasis and distant metastasis.
