# Supplementary material for: Hsa_circ_0005273 facilitates breast cancer tumorigenesis by regulating YAP1-hippo signaling pathway
Source: J Exp Clin Cancer Res. 2021 Jan 12;40:29. doi: 10.1186/s13046-021-01830-z (PMC7802350; doi:10.1186/s13046-021-01830-z)
Supplement: Supplementary file 5 — Additional file 5 Table S4 The relationship between the expression of hsa_circ_0005273 and various clinicopathological variables of luminal-like cohort in BC patients. [file 13046_2021_1830_MOESM5_ESM.docx]

| Patients Characteristics | Total | hsa_circ_0005273 expression | |  |
| --- | --- | --- | --- | --- |
|  |  | High (*N=37)* | Low (*N=19)* | P value* |
| Age |  |  |  | 0.4001 |
| <60 | 25 | 18 | 7 |  |
| ≥60 | 31 | 19 | 12 |  |
| TNM stage |  |  |  | 0.2217 |
| Ⅰ and Ⅱ | 32 | 19 | 13 |  |
| Ⅲ and Ⅳ | 24 | 18 | 6 |  |
| Tumor size(cm) |  |  |  | 0.3047 |
| ≤2 | 46 | 29 | 17 |  |
| ＞2 | 10 | 8 | 2 |  |
| Lymph node metastasis |  |  |  | 0.4181 |
| negative | 47 | 30 | 17 |  |
| positive | 9 | 7 | 2 |  |
| Distant metastasis |  |  |  | 0.3021 |
| No | 54 | 35 | 19 |  |
| Yes | 2 | 2 | 0 |  |

**Table S4** **The relationship between the expression of hsa_circ_0005273 and various clinicopathological variables of luminal-like cohort in BC patients.**

**Table S4** The relationship between the expression of hsa_circ_0005273 and various clinicopathological variables of luminal-like cohort in BC patients (n=56). High expression of hsa_circ_0005273 had no correlation with various clinicopathological variables.
