# Supplementary material for: Hsa_circ_0005273 facilitates breast cancer tumorigenesis by regulating YAP1-hippo signaling pathway
Source: J Exp Clin Cancer Res. 2021 Jan 12;40:29. doi: 10.1186/s13046-021-01830-z (PMC7802350; doi:10.1186/s13046-021-01830-z)

Original western blotting images in Fig. 2J

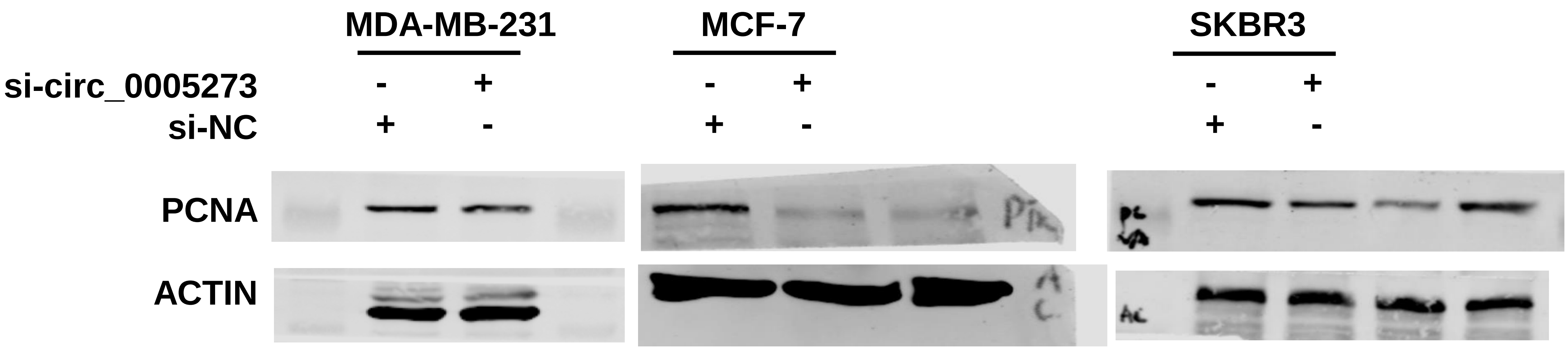

Original western blotting images in Fig. 4J

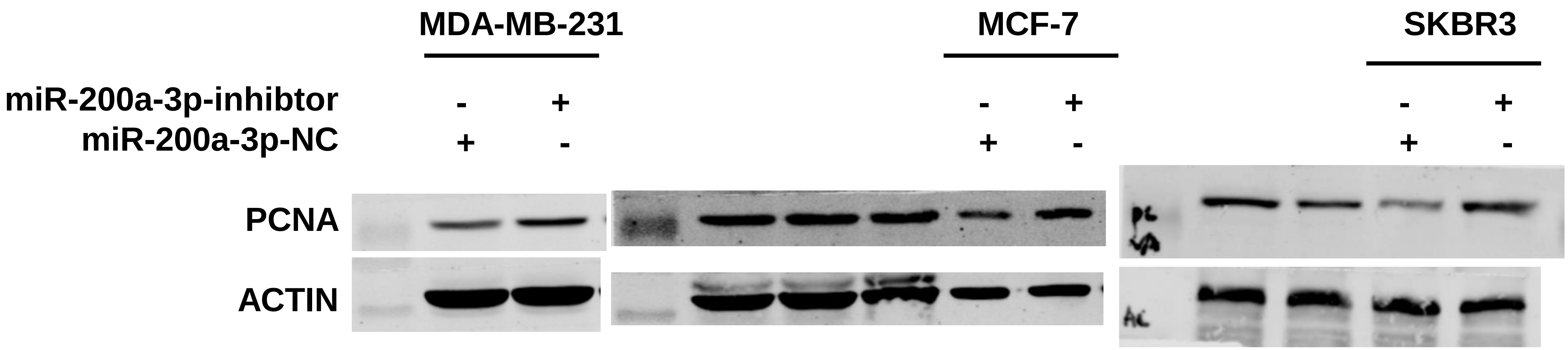

Original western blotting images in Fig. 5H

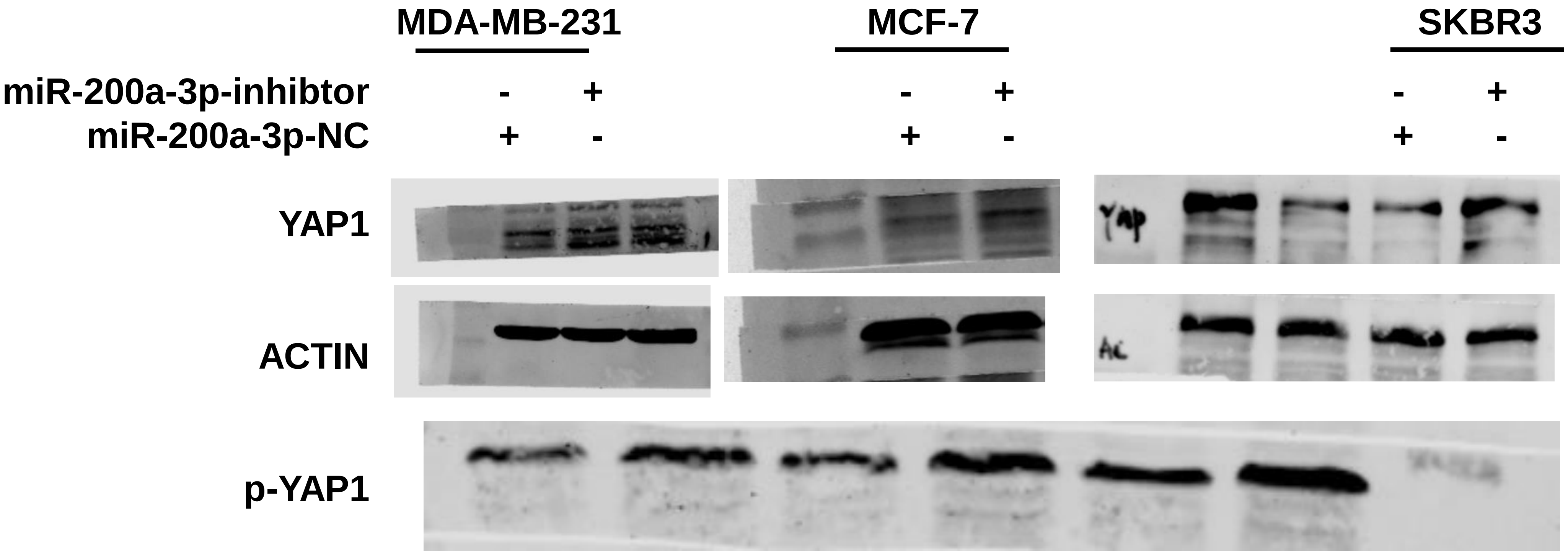

Original western blotting images in Fig. 5J

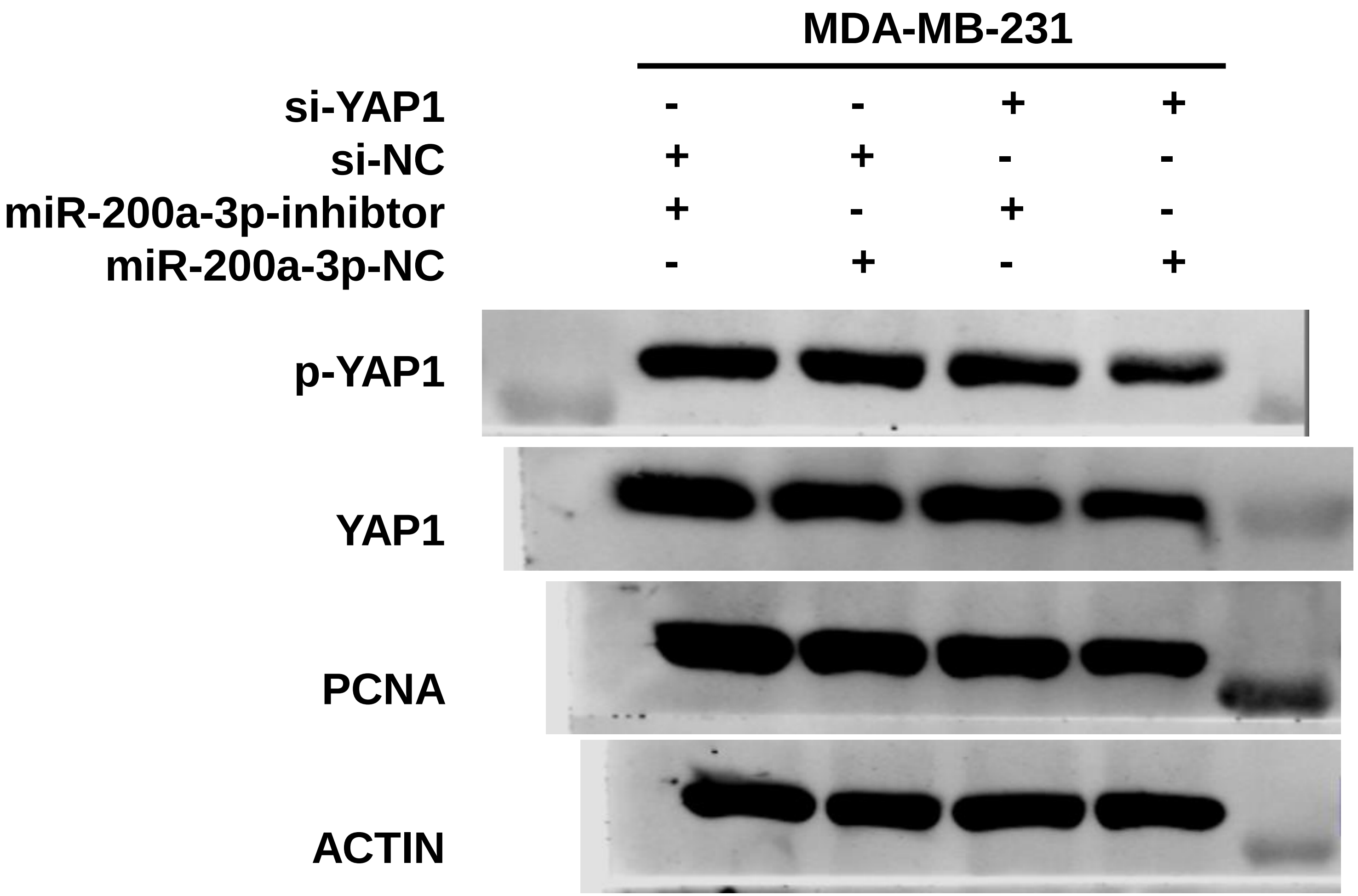

Original western blotting images in Fig. 5L

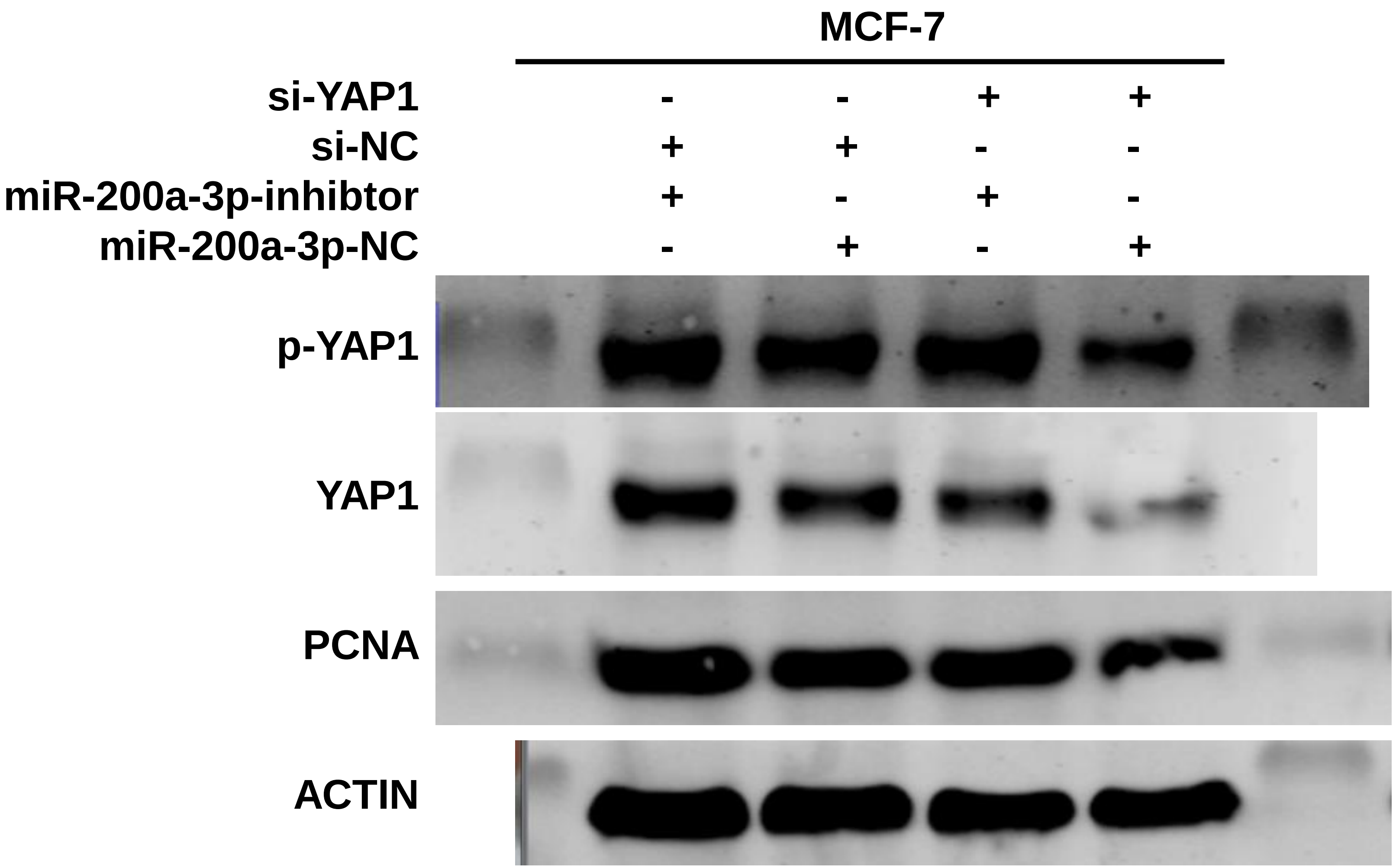

Original western blotting images in Fig. 5N

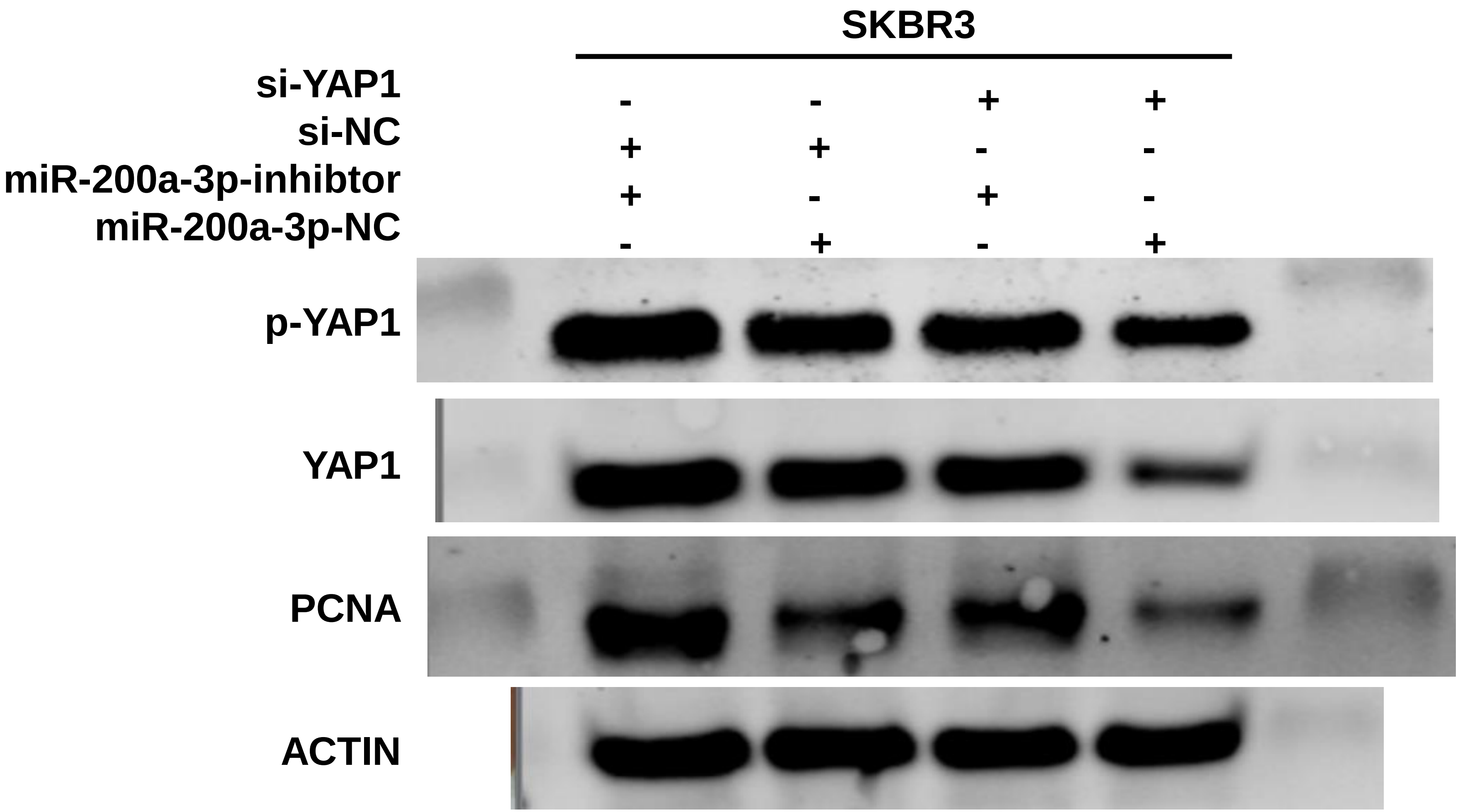

Original western blotting images in Fig. 6D

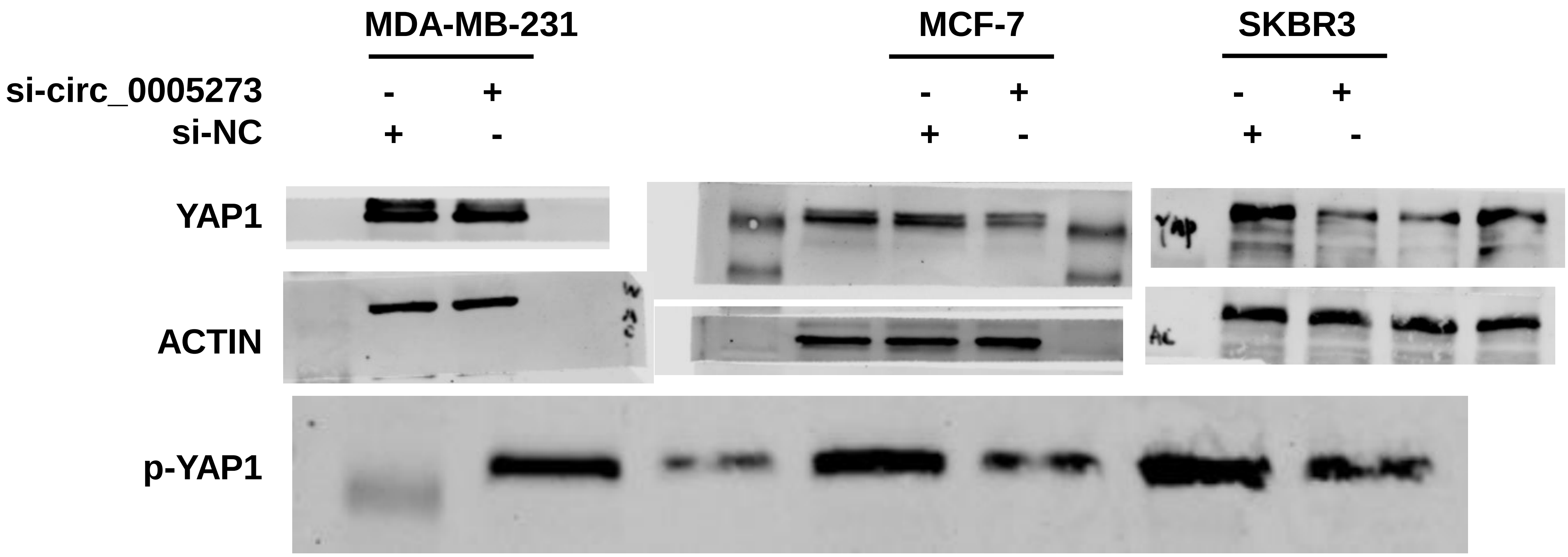

Original western blotting images in Fig. 6L

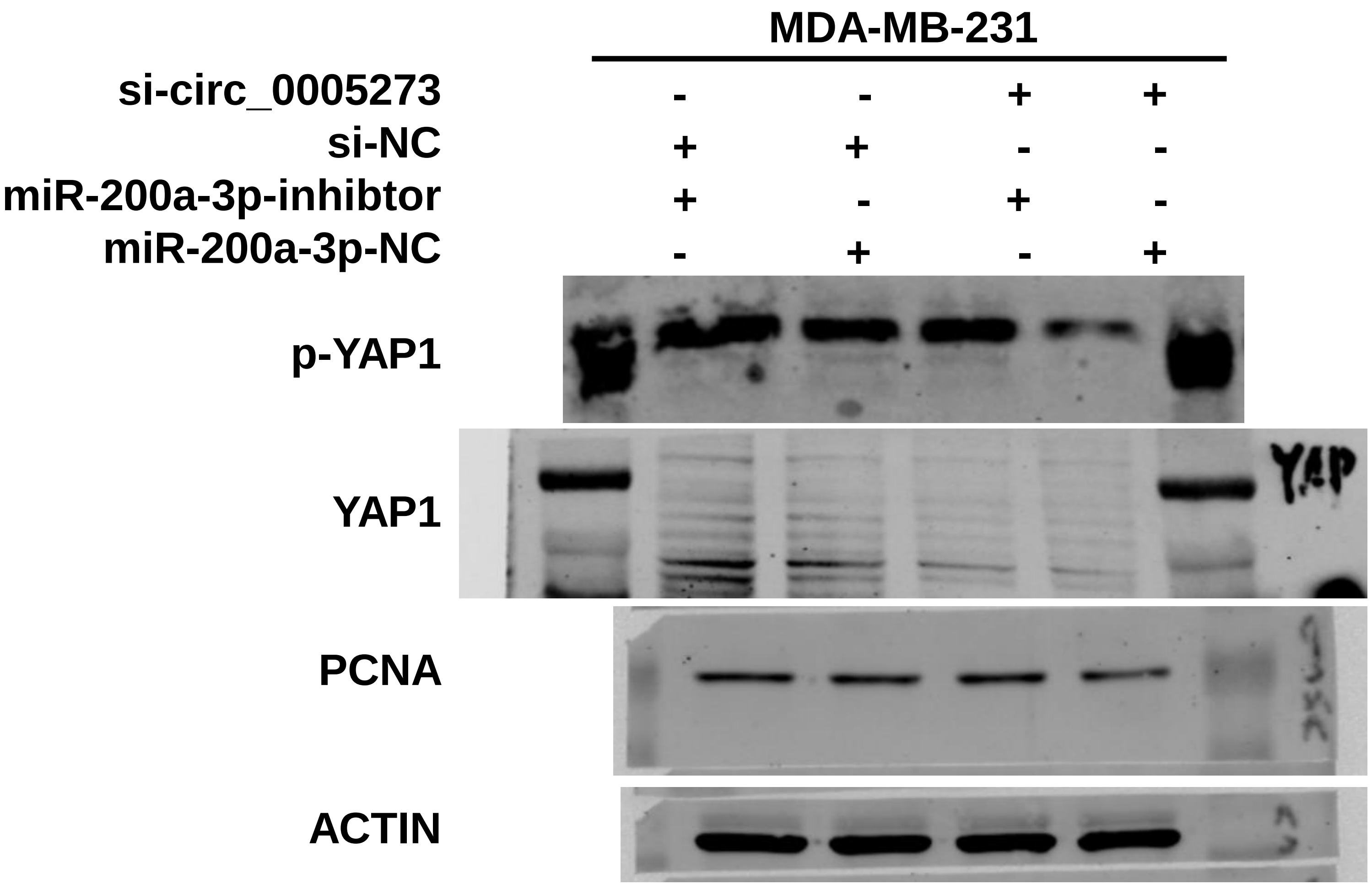

Original western blotting images in Fig. 6M

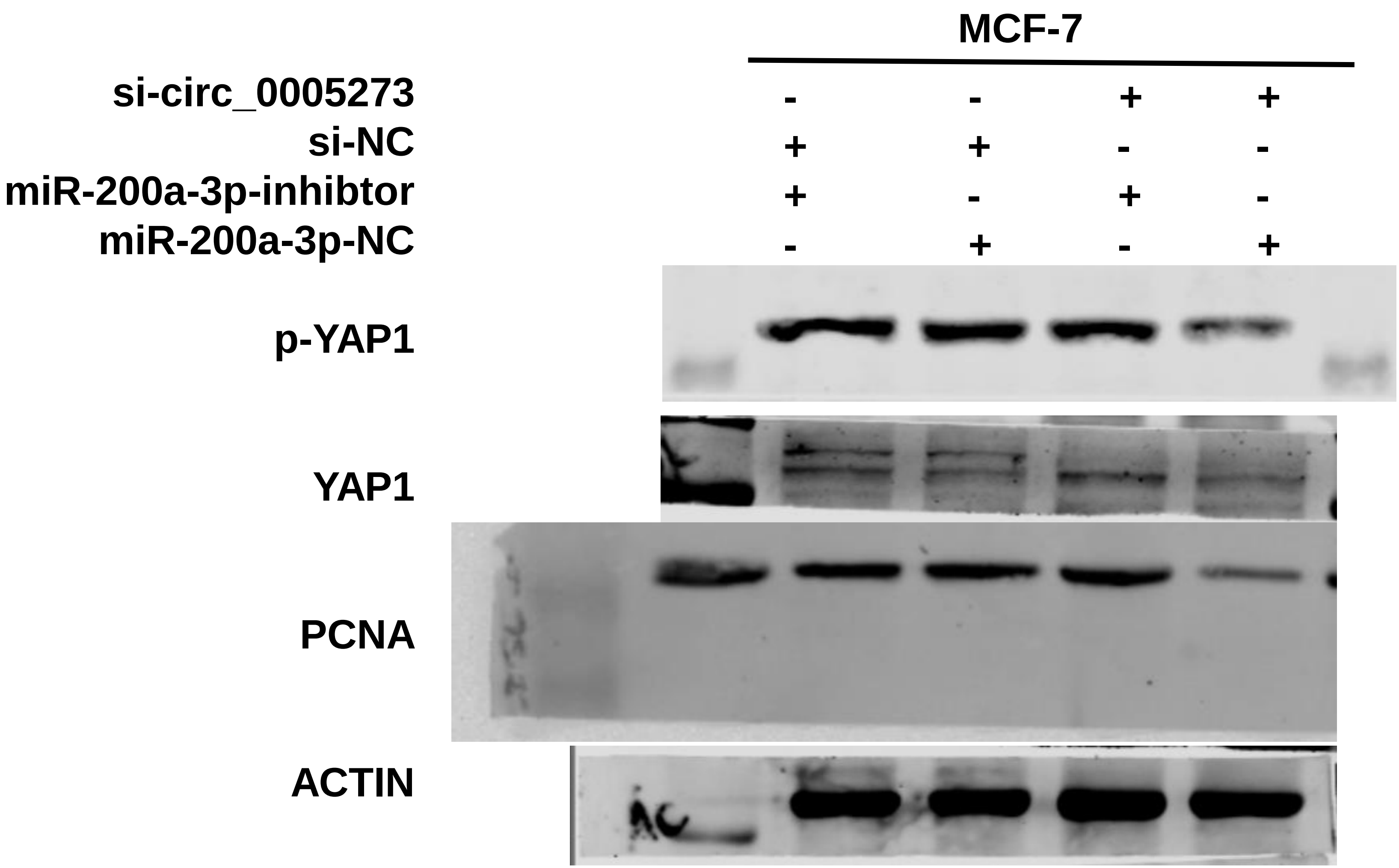

Original western blotting images in Fig. 6N

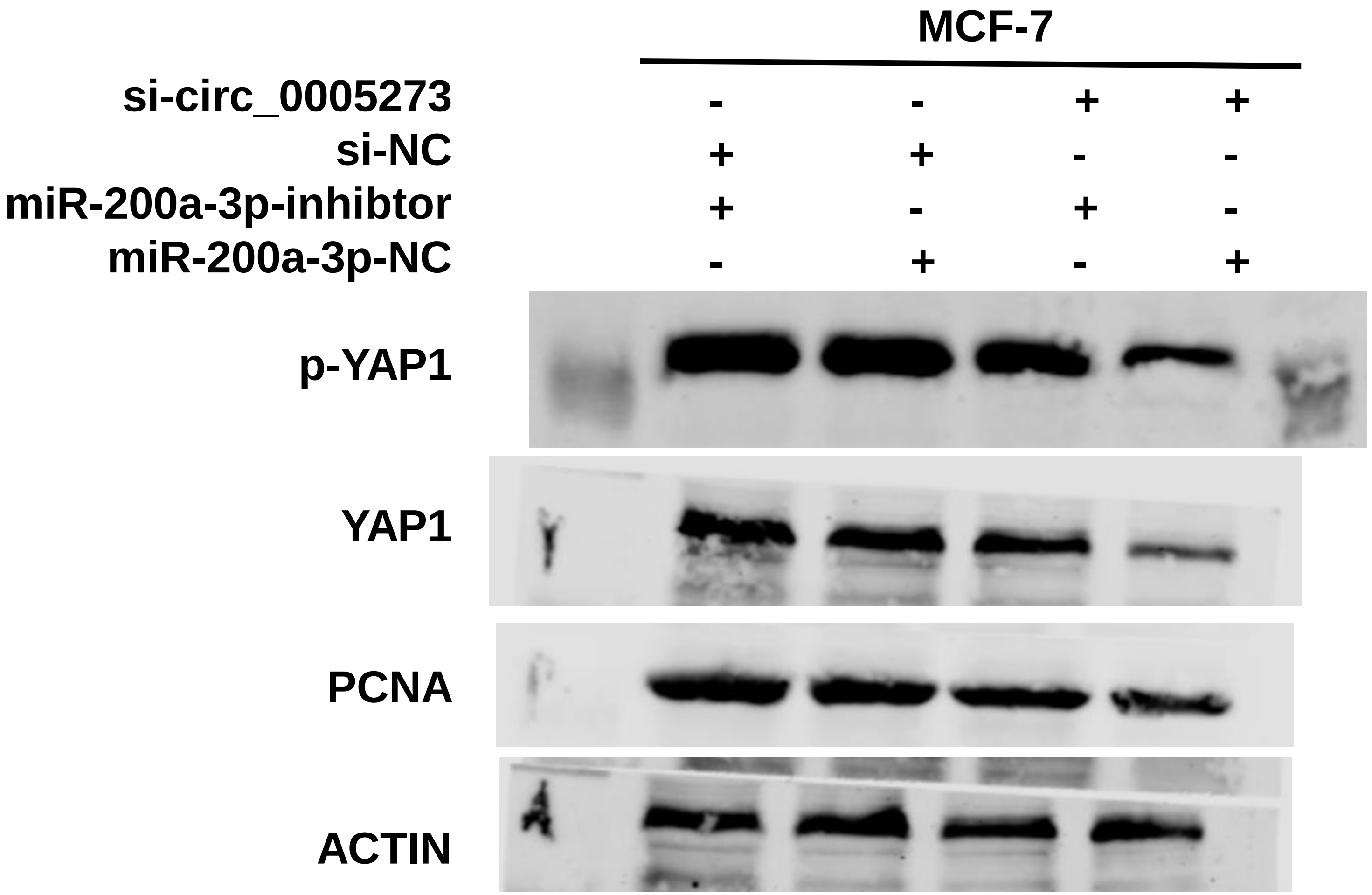

Original western blotting images in Fig. 7H

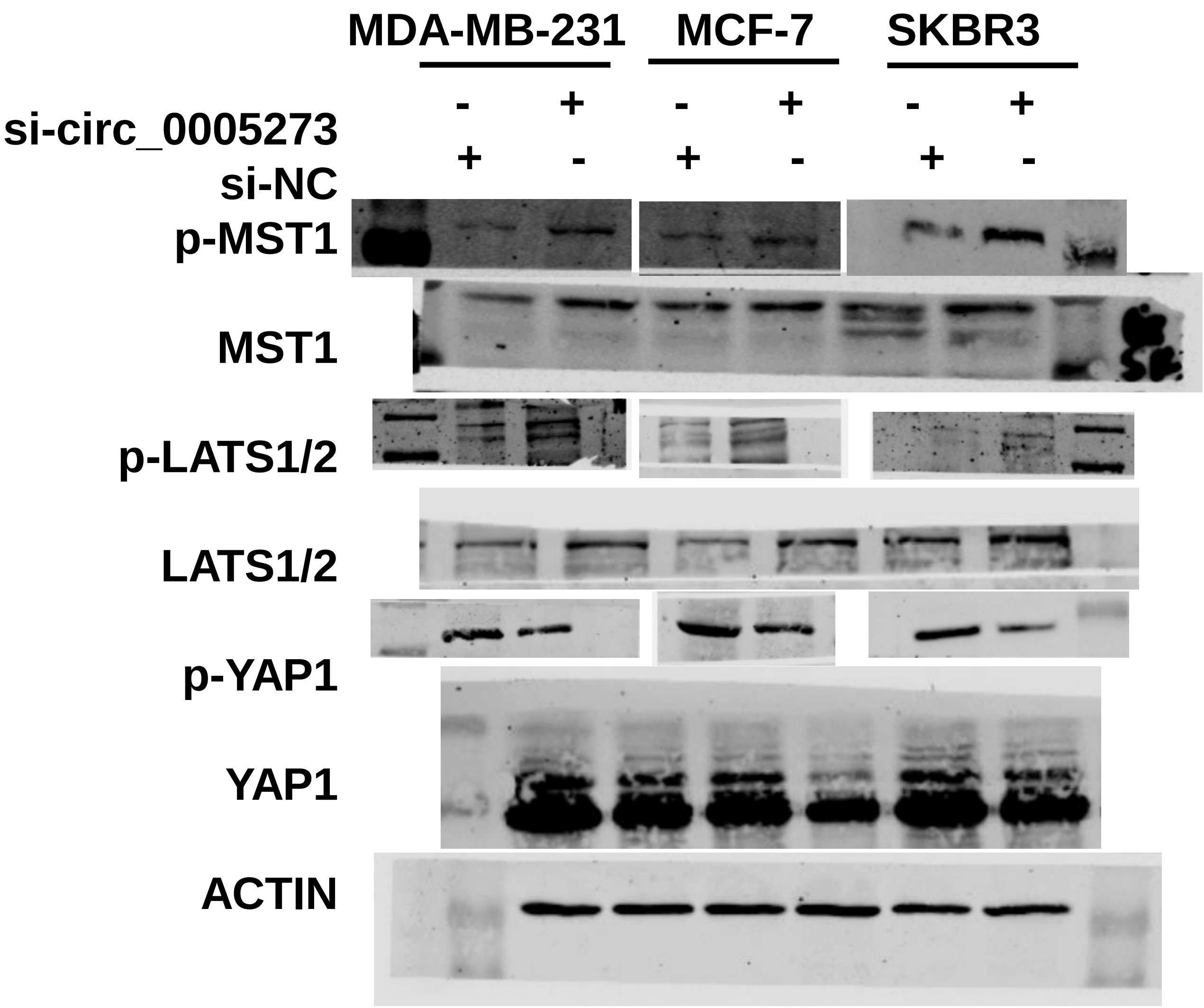

Original western blotting images in Fig. 7I

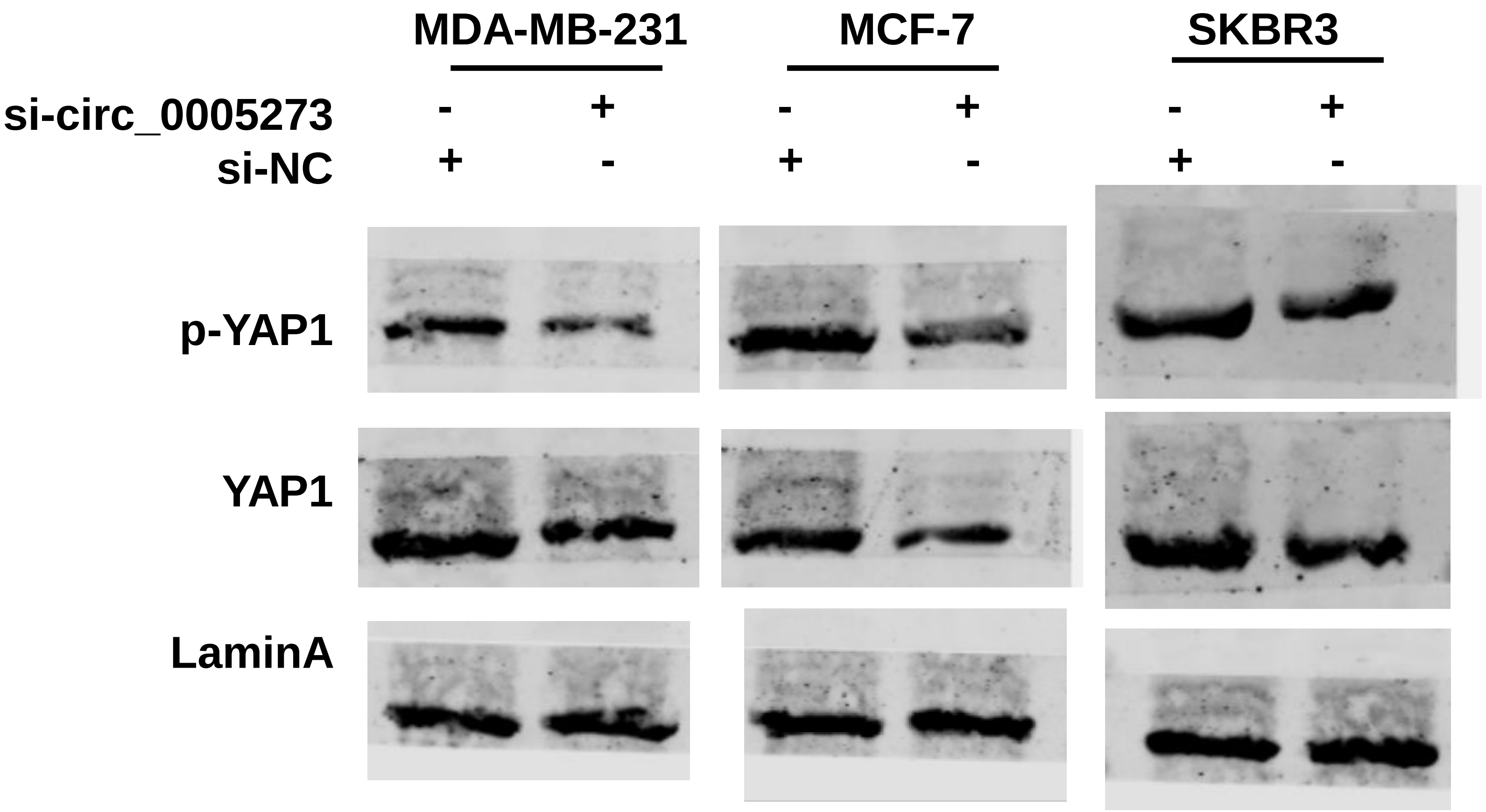

Original western blotting images in Fig. 7K

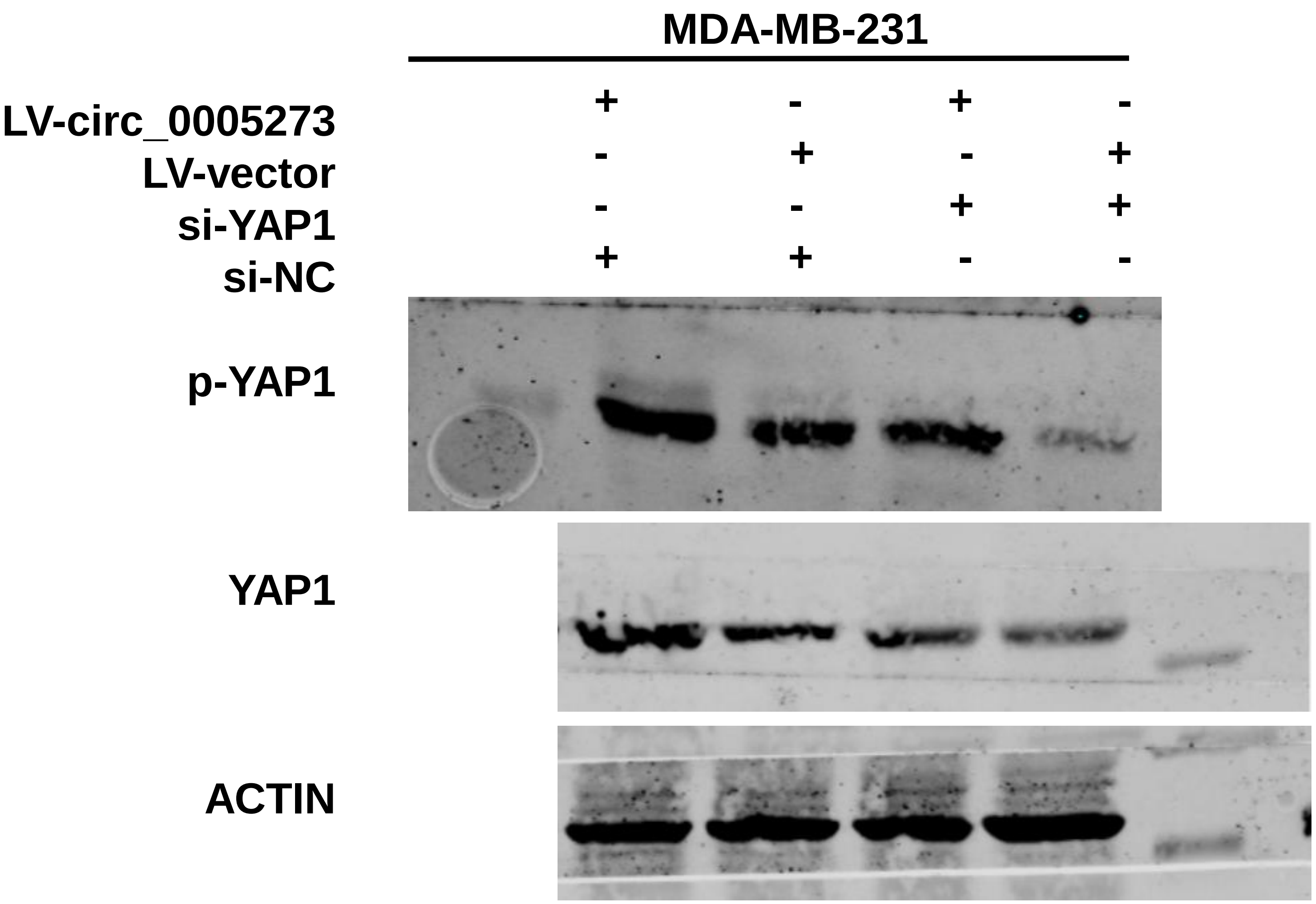

Original western blotting images in Fig. 7M

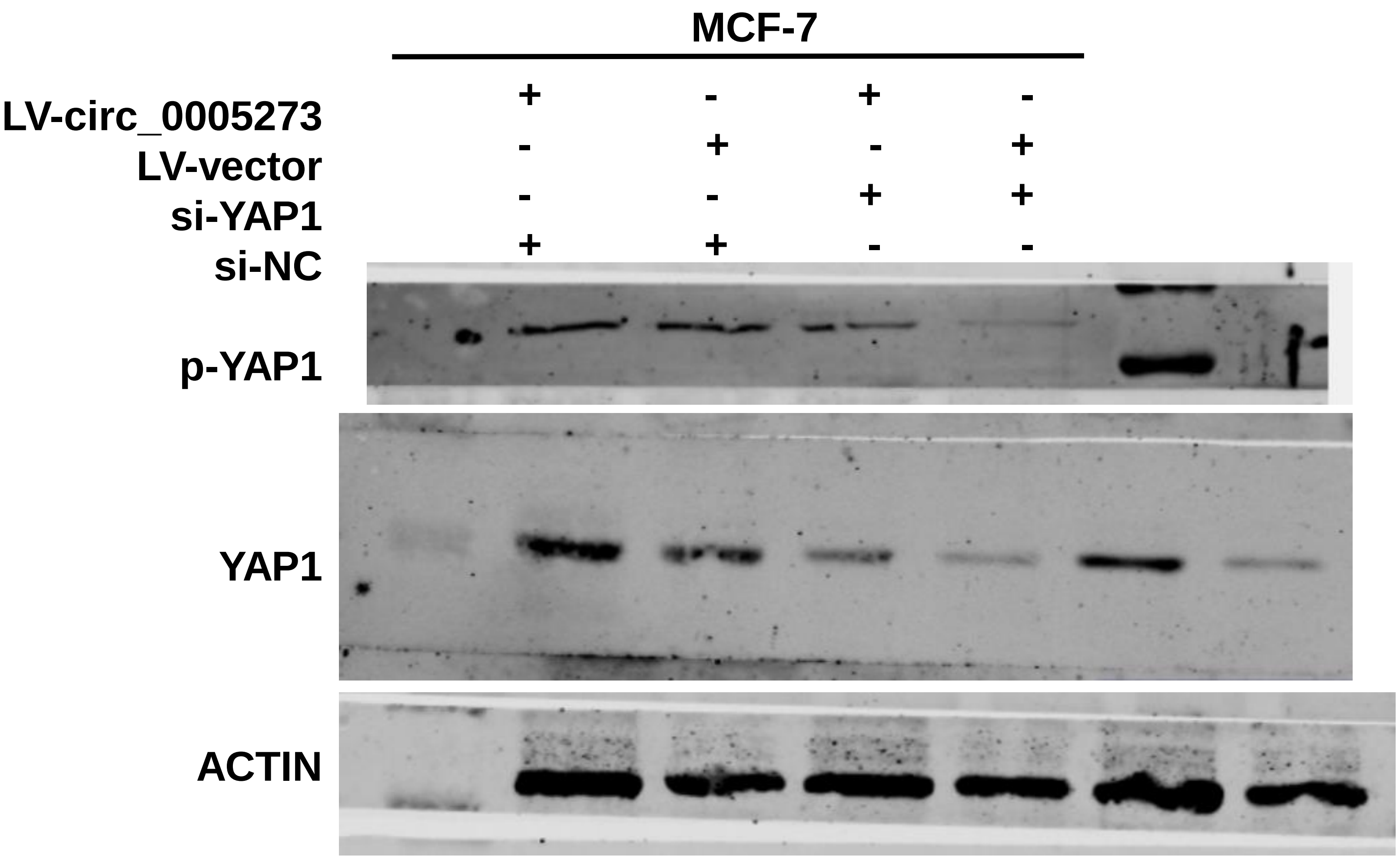

Original western blotting images in Fig. 7O

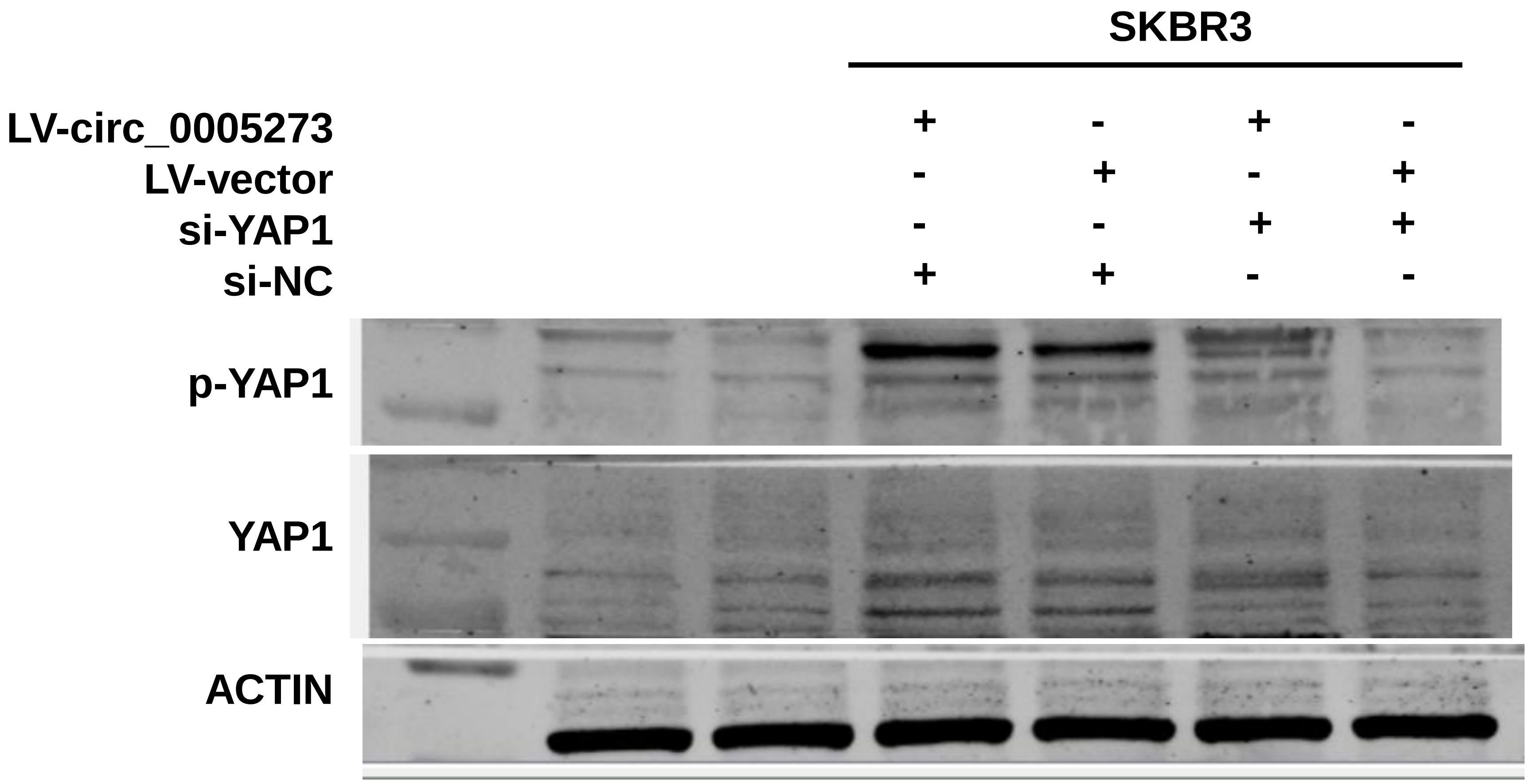

Original western blotting images in Fig. 8F

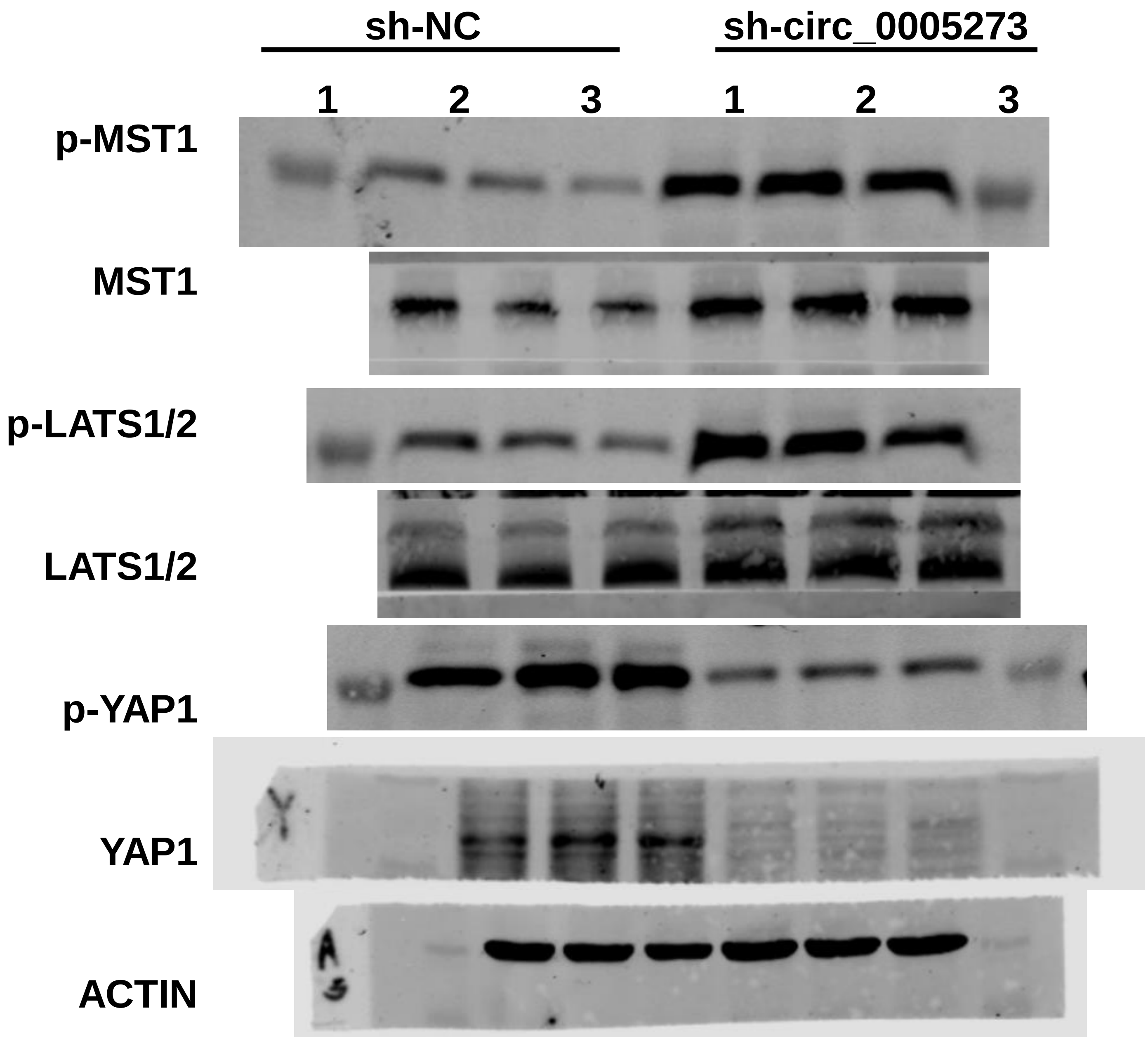

Original western blotting images in Fig. S1D

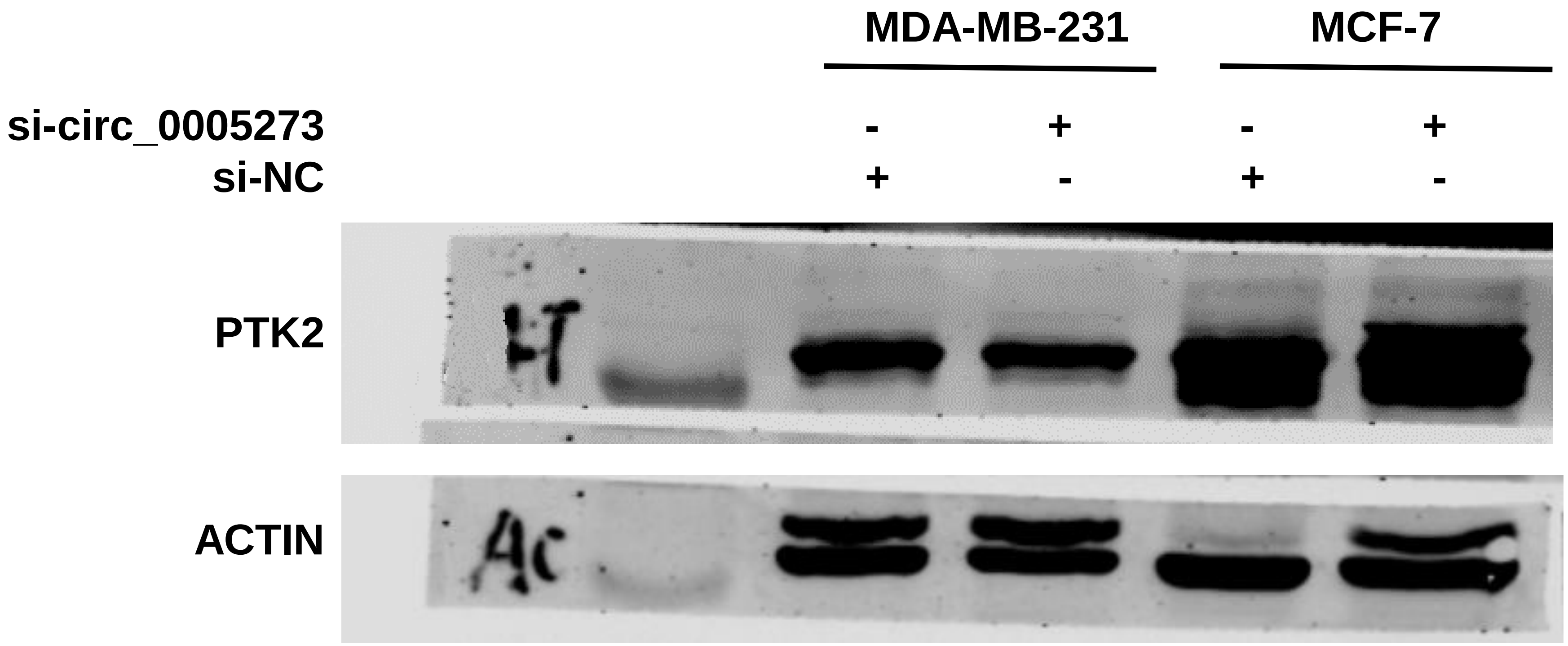

Original western blotting images in Fig. S1E

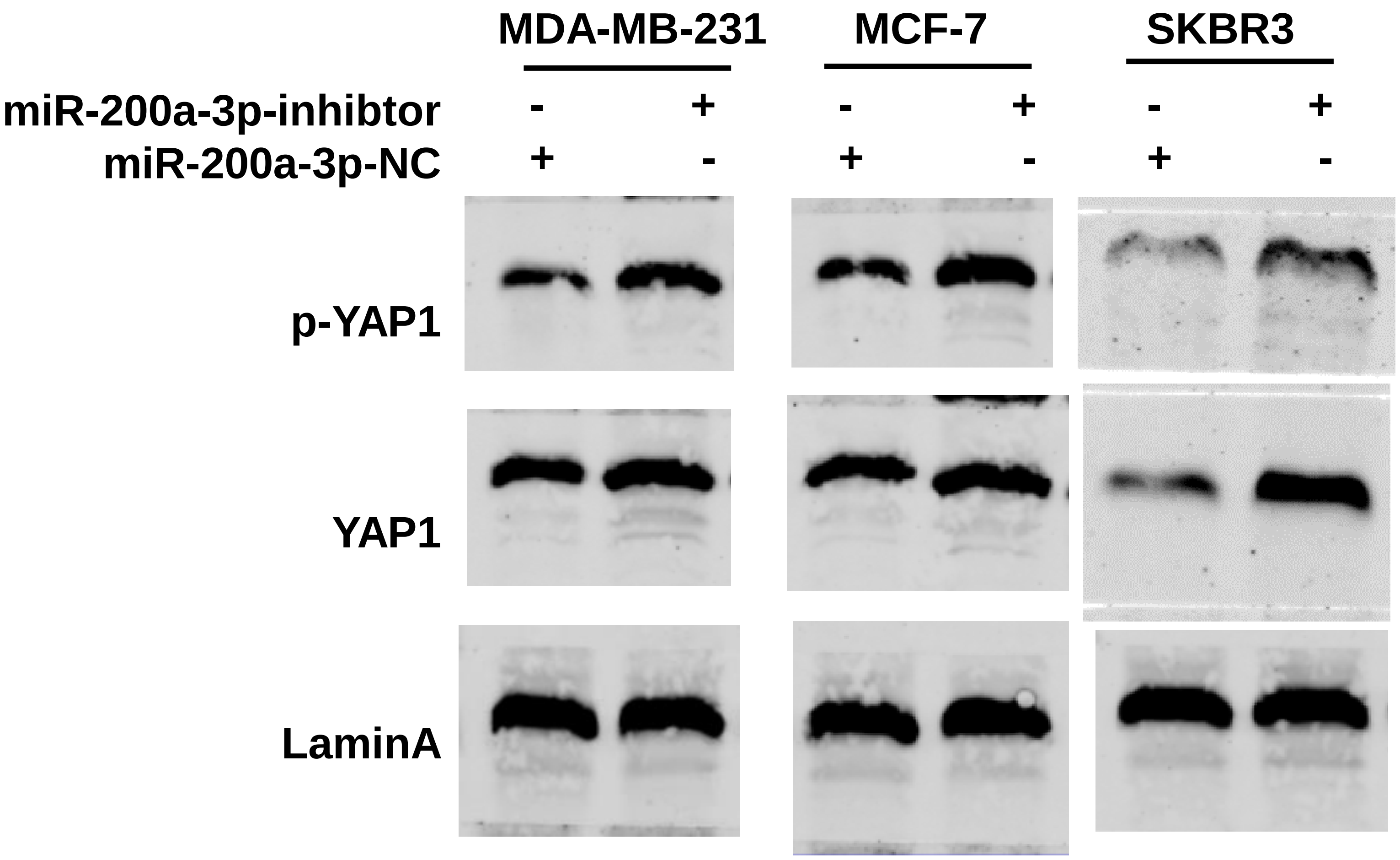

Supplement: Supplementary file 6 — Additional file 6. Original western blotting images in this study. [file 13046_2021_1830_MOESM6_ESM.pdf]
